# Effect of managed transition on mental health outcomes for young people at the child–adult mental health service boundary: a randomised clinical trial

**DOI:** 10.1017/S0033291721003901

**Published:** 2023-04

**Authors:** S. P. Singh, H. Tuomainen, G. Bouliotis, A. Canaway, G. De Girolamo, G. C. Dieleman, T. Franić, J. Madan, A. Maras, F. McNicholas, M. Paul, D. Purper-Ouakil, P. Santosh, U. M. E. Schulze, C. Street, S. Tremmery, F. C. Verhulst, P. Wells, D. Wolke, J. Warwick, the MILESTONE Consortium, Swaran Singh, Helena Tuomainen, Jason Madan, Moli Paul, Cathy Street, Dieter Wolke, Jane Warwick, Claire Daffern, Paramala Santosh, Federico Fiori, Giovanni de Girolamo, Diane Purper-Ouakil, Athanasios Maras, Frank Verhulst, Gwen C Dieleman, Ulrike Schulze, Andrea Wohner, Sabine Tremmery, Fiona McNicholas, Tomislav Franić, Maryann Davis, Pat McGorry, Adriana Mihai, Norman Sartorius, Priya Tah, Alastair Canaway, James Griffin, Rebecca Appleton, Philip Wells, Natalie Heaney, Kate Lievesley, Mathilde Mastroianni, Jatinder Singh, Laura Adams, Giulia Signorini, Alessandro Ferrari, Elisa Gheza, Cecilia Ferrari, Laura Rivolta, Flavia Levi, Maria Cataldo, Lidia Manenti, Giorgia Morini, Adriana Pastore, Pamela Stagni, Cecilia Toselli, Pamela Varvara, Frédérick Russet, Virginie Maurice, Véronique Humbertclaude, Larissa S van Bodegom, Mathilde M Overbeek, Suzanne E Gerritsen, Melanie Saam, Ulrike Breuninger, Gaëlle Hendrickx, Veronique De Roeck, Ingrid Holme, Aleksandra Gronostaj, Rachael McKenna, Lesley O'Hara, Nikolina Davidović, Helena Tomljenovic, Amanda Tuffrey, Anna Wilson, Charlotte Gatherer, Leanne Walker, Jo Berriman, Vinuthna Pemmaraju, Michael Slowik, Ben Rogers, Alan Farmer, Paramala Santosh, Giovanni Allibrio, Marco Armandi, Angelo Bertani, Giuseppe Carrà, Patrizia Conti, Sabrina Ferrari, Francesco Margari, Ottaviano Martinelli, Renata Nacinovich, Paolo Scocco, Paolo Stagi, Fabio Vanni, Stefano Vicari, Mario Speranza, Hélène Lida-Pulik, Renaud Jardri, Aesa Parenti, David Da Fonseca, Isabelle Charvin, Frédérique Bonnet-Brilhault, Chrystèle Bodier, Catherine Prigent, Aurélie Schandrin, Therese van Amelsvoort, Albert Boon, Arno van Dam, Jörg M. Fegert, Renate Schepker, Elena Tanase, Beata Williams, Michele Noterdaeme, Anne Sartor, Vehbi Sakar, Caroline von Bentzel, Emmanuel Nelis, Dirk van West, Catherine Klockaerts, Ann Pepermans, Marina Danckaerts, Anne Roekens, Hanne Van Gutschoven, Christel Lippens, Jean-Pierre Ermans, Helen Keeley, Keith Holmes, James McDonald, Maria Migone, Fionnuala Lynch, Elaine Healy, Peter Dineen, Vlatka Kovač, Katarina Dodig-Ćurković

**Affiliations:** 1 Mental Health and Wellbeing, Warwick Medical School, University of Warwick, Coventry, UK; 2 Coventry and Warwickshire Partnership NHS Trust, Coventry, UK; 3 Warwick Clinical Trials Unit, Warwick Medical School, University of Warwick, Coventry, UK; 4 IRCCS Istituto Centro San Giovanni di Dio Fatebenefratelli, Brescia, Italy; 5 Department of Child and Adolescent Psychiatry and Psychology, Erasmus Medical Center, Rotterdam, the Netherlands; 6 Department of Psychiatry, Clinical Hospital Center Split, Split, Croatia; 7 Yulius Academy, Rotterdam, the Netherlands; 8 Department of Child and Adolescent Psychiatry, University College Dublin School of Medicine and Medical Science, Dublin, Republic of Ireland; 9 Geary Institute, University College Dublin, Dublin, Republic of Ireland; 10 Department of Child Psychiatry, Our Lady's Hospital for Sick Children, Dublin, Republic of Ireland; 11 Lucena Clinic SJOG, Dublin, Republic of Ireland; 12 Centre Hospitalier Universitaire de Montpellier, Saint Eloi Hospital, Unit of Child and Adolescent Psychiatry (MPEA1), Montpellier, France; 13 Department of Child and Adolescent Psychiatry, Institute of Psychiatry, Psychology and Neuroscience, King's College London, London, UK; 14 Centre for Interventional Paediatric Psychopharmacology and Rare Diseases (CIPPRD), National and Specialist Child and Adolescent Mental Health Services, Maudsley Hospital, London, UK; 15 HealthTracker Ltd, Gillingham, UK; 16 Department of Child and Adolescent Psychiatry/Psychotherapy, University of Ulm, Ulm, Germany; 17 Department of Neurosciences, Child & Adolescent Psychiatry, University of Leuven, Leuven, Belgium; 18 Department of Clinical Medicine, University of Copenhagen, Copenhagen, Denmark; 19 Department of Psychology, University of Warwick, Coventry, UK

**Keywords:** Transition, adolescent, mental disorders, mental health services, structured assessment, cluster randomised controlled trial, HoNOSCA

## Abstract

**Background:**

Poor transition planning contributes to discontinuity of care at the child–adult mental health service boundary (SB), adversely affecting mental health outcomes in young people (YP). The aim of the study was to determine whether managed transition (MT) improves mental health outcomes of YP reaching the child/adolescent mental health service (CAMHS) boundary compared with usual care (UC).

**Methods:**

A two-arm cluster-randomised trial (ISRCTN83240263 and NCT03013595) with clusters allocated 1:2 between MT and UC. Recruitment took place in 40 CAMHS (eight European countries) between October 2015 and December 2016. Eligible participants were CAMHS service users who were receiving treatment or had a diagnosed mental disorder, had an IQ ⩾ 70 and were within 1 year of reaching the SB. MT was a multi-component intervention that included CAMHS training, systematic identification of YP approaching SB, a structured assessment (Transition Readiness and Appropriateness Measure) and sharing of information between CAMHS and adult mental health services. The primary outcome was HoNOSCA (Health of the Nation Outcome Scale for Children and Adolescents) score 15-months post-entry to the trial.

**Results:**

The mean difference in HoNOSCA scores between the MT and UC arms at 15 months was −1.11 points (95% confidence interval −2.07 to −0.14, *p* = 0.03). The cost of delivering the intervention was relatively modest (€17–€65 per service user).

**Conclusions:**

MT led to improved mental health of YP after the SB but the magnitude of the effect was small. The intervention can be implemented at low cost and form part of planned and purposeful transitional care.

## Introduction

Transition from paediatric to adult care is problematic in many health specialities, but most severe, complex and challenging in mental health care (Singh, Anderson, Liabo, & Ganeshamoorthy, 2016). It should be a planned and purposeful process which addresses the needs of young people (YP) as they move to adult services and towards independence (Paul et al., 2013). Previous studies suggest that transition is ‘poorly planned, poorly executed, and poorly experienced’ (Singh et al., 2010). Many YP experience discontinuity or disengagement from care on reaching the child–adult mental health service boundary (SB) (Appleton, Connell, Fairclough, Tuomainen, & Singh, 2019; Leavey et al., 2019; Singh et al., 2010), with potentially adverse impact on their health and wellbeing (Davis, Koroloff, Sabella, & Sarkis, 2018). Barriers to optimal transition between child/adolescent mental health service (CAMHS) and adult mental health service (AMHS), although well-documented, are complex to overcome (Hovish, Weaver, Islam, Paul, & Singh, 2012). Core elements needed to improve transition are implementing policy, tracking and monitoring transition readiness, transition planning, transfer of care, and completion (Cleverley, Rowland, Bennett, Jeffs, & Gore, 2020; Singh et al., 2016). There is as yet no standardised, shared or robustly tested model of transitional care in the published literature (Singh et al., 2016; Tuomainen, Appleton, & Singh, 2020). MILESTONE was an eight-country 5-year EU-funded project to improve the experiences of YP at the child/adolescent to AMHS interface (Tuomainen et al., 2018). The project comprised of nine distinct but inter-related work packages, which have been described elsewhere (Singh et al., 2017; Tuomainen et al., 2018). We created a Transition Readiness and Appropriateness Measure (TRAM) (Santosh et al., 2020b), which systematically identifies the needs, preferences and readiness of YP for ongoing adult care, including those who can be appropriately discharged. Based on the TRAM, we developed a model of transitional care, managed transition (MT) (online Supplementary Fig. S1) (Singh et al., 2017). We conducted a cluster-randomised trial to assess the effect of MT on mental health outcomes of YP approaching the SB of their CAMHS compared with usual care (UC) and a within-trial economic evaluation to estimate the cost-effectiveness of MT.

## Methods

### Design

We conducted an eight-country, two-arm, parallel design, superiority, cluster-randomised controlled trial (cRCT) to assess whether MT improves mental health outcomes in YP approaching the CAMHS SB, compared to UC. The cRCT with economic evaluation was embedded within the MILESTONE longitudinal study (ISRCTN83240263 and NCT03013595), described elsewhere and funded by the European Union (Singh et al., 2017). Participating countries were Belgium, Croatia, France, Germany, Ireland, Italy, Netherlands and the UK. The authors assert that all procedures contributing to this study comply with the ethical standards of the relevant national and institutional committees on human experimentation and with the Helsinki Declaration of 1975, as revised in 2008. All procedures involving human subjects were approved in the UK by the National Research Ethics Service Committee West Midlands – South Birmingham (15/WM/0052) and equivalent ethics boards in the participating countries. The study protocol has been published (Singh et al., 2017).

### Trial setting, eligibility and participants

Participating CAMHS were organisations delivering medical and psychosocial interventions for children/adolescents with mental health and/or neuropsychiatric/developmental disorders. Services could be publicly or privately funded but had to have a formal upper age limit (the SB) for providing care. Forensic services and highly specialised services were excluded. The child/adolescent to adult mental health SB was typically 18 years but varied according to local practice (online Supplementary Table S2). Eligible participants were YP with a mental disorder defined by DSM-IV-TR, DSM-5 or ICD 10/11, who were receiving CAMHS care, with an IQ ⩾ 70 and within 1 year of reaching CAMHS SB. Recruitment occurred between 1st October 2015 and 31st December 2016. Because transition decisions were sometimes made after the YP reached the SB, we amended the eligibility criteria (in April 2016) to include YP who were up to 3-months older than the SB.

Participants were identified by clinicians, and other care staff, and recruited by local study personnel. The YP's parent (legal guardian, a partner, an older adult sibling or another individual) was also invited to participate, provided the young person agreed. Written informed consent was obtained from all participants. For YP below the age of consent (UK <16, Europe <18), a parent/legal guardian was required to provide consent, with the YP signing an assent form. YP involved in the study were offered incentives as ‘token of thanks’ for participating. Incentives were shopping vouchers (in all countries but Italy and Croatia) and/or being entered into a prize draw (Singh et al., 2017). Further details of methodology and design have been reported elsewhere (Singh et al., 2017).

### Randomisation and masking

Clusters were individual CAMHS recruited to the MILESTONE study (cRCT, economic evaluation and longitudinal cohort study) (Singh et al., 2017). Randomisation (2:1 between UC and MT) was undertaken in two stages and stratified by country to ensure that the number of clusters per country was divisible by 3 and that all countries had both control and intervention clusters. It was not feasible to blind clinicians or the research team, but YP and parents/carers were informed of trial arm, only if requested, after consenting to participate.

### Trial interventions

MT (online Supplementary Fig. S1) was a multi-component intervention that included CAMHS training, systematic identification of YP approaching SB, a structured assessment (TRAM) and feedback of the TRAM findings to the CAMHS clinician (via the TRAM summary report). Prior to opening to recruitment, CAMHS clinicians at the intervention sites were invited to a single standardised training session on good/effective transition and interpretation of the TRAM summary report. Once the TRAM summary report relating to a participant (online Supplementary Fig. S2) was ready, it was sent to the CAMHS clinician along with a letter advising that it should be discussed with the YP and parent/carer and attached to any referral letter to AMHS (online Supplementary Fig. S3). To reinforce the learning undertaken during training, a leaflet reminding the CAMHS clinician why care in transition is important, and what YP say helps them with transition, was also included (online Supplementary Fig. S4). Clinicians in the UC group did not receive the TRAM summary report or any training regarding transition.

### Data collection

Outcomes were assessed at baseline, 9 months and 15 months using structured interviews (by research assistants not blinded to group membership) and self-reported measures (online Supplementary Table S3). Data were collected via a web-based, secure, data capture system (HealthTracker™). Interviews to complete the Health of the Nation Outcome Scale for Child and Adolescents (HoNOSCA) were held at the CAMHS, the participant's home, or an alternative location (according to the participant's preference). Participants were asked to complete other measures within 2 weeks of the HoNOSCA interview but were allowed a 3-month window.

### Outcomes

#### Primary outcome

The primary outcome was HoNOSCA score 15-months post-entry to the trial (Garralda, Yates, & Higginson, 2000; Gowers et al., 1999). This validated and internationally widely implemented global outcome measure for child/adolescent mental health care includes domains of symptoms, behaviour, impairments and social functioning (Harris et al., 2018). Items 1–13 are scored on a scale of 0–4 (0 indicates no problem, 4 severe problem; a detailed glossary describes scales for each item) (Ballesteros-Urpí et al., 2018; Harris et al., 2018). The total score, indicating the severity of the mental health problem/s, is the sum of the 13 items. HoNOSCA was completed by a trained Research Assistant following semi-structured interviews with the young person, and (where possible) with parent/carer and relevant clinician (or review of medical records if the relevant clinician was not available).

#### Secondary outcomes

Secondary outcomes were self-reported HoNOSCA score (HoNOSCA-SR) (Gowers, Levine, Bailey-Rogers, Shore, & Burhouse, 2002), transition outcomes (TROM) (Santosh et al., 2020a), quality of life (WHOQOL-BREF) (Skevington, Lotfy, & O'Connell, 2004), functioning and impairment (SLOF) (Mucci et al., 2014), self-reported emotional/behaviour problems (YSR/ASR) (Achenbach & Rescorla, 2001), parent/carer reported emotional/behavioural problems (CBCL/ABCL) (Achenbach & Rescorla, 2003), independent behaviour (IBDCS) (van Staa, 2011), illness severity (CGIS) (Guy, 1976), transfer experience (OYOF-TES) (van Staa & Sattoe, 2014), health-related quality of life (EQ-5D-5L) (Herdman et al., 2011) and resource utilisation (CSRI) (Chisholm et al., 2000). Further details on outcome measures are given in online Supplementary Table S4. Information on YP's referral and transition status was collected from their CAMHS clinician. Information on severe adverse events (SAEs) was collected, specifically self-harm, suicidal thoughts, suicide through physical harm or with drugs, or death.

### Statistical analysis

Assuming an average cluster size of 15 participants, an allocation ratio of 2:1 (control:intervention), a coefficient of variation of cluster size of 0.4 (cluster size ranging from 5 to 30), and an intra-cluster correlation coefficient of 0.01, we estimated that with 600 participants [195 intervention arm (13 clusters), 405 control arm (27 clusters)] the trial would have 89% power to detect a difference of 0.30 standard deviations (s.d.s) in the primary outcome measure (HoNOSCA). Allowing for 30% attrition, we aimed to recruit 21 participants per cluster. The target sample size was, therefore, 840 participants in total (273 intervention; 567 control).

We tested the null hypothesis of no difference in mean HoNOSCA score between the MT and UC groups at month 15 post-randomisation, using a generalised linear mixed effect model (GLMM) with random and fixed effects (to allow for the hierarchical structure of the data and adjust for trial arm, time point and baseline characteristics, HoNOSCA score, gender and diagnosis). Secondary outcomes HoNOSCA-SR and quality of life (WHOQOL-BREF) were analysed using a hierarchical linear mixed model with random effects for the four levels (country, site, participant and follow-up time) and adjustment for the same fixed effects. For other secondary outcomes, descriptive analyses were conducted. The significance level was set at 5%, with no adjustment for multiple testing. All analyses were on an intention-to-treat basis and followed a pre-specified statistical analysis plan (available at https://www.milestone-transitionstudy.eu/).

To examine the cost of implementing the intervention, clinicians were invited to complete a questionnaire on the time burden of completing TRAM. Time spent by the Warwick team preparing and checking TRAM reports was also recorded. We also captured the resources required to set up the intervention and conduct initial training of clinicians. Resource use data were combined with unit cost data (see online Supplementary appendix, ‘Intervention costing’) to estimate intervention costs.

All analyses were conducted using Stata 16 (StataCorp, 2019).

## Results

### Participants

**Fig. 1.**
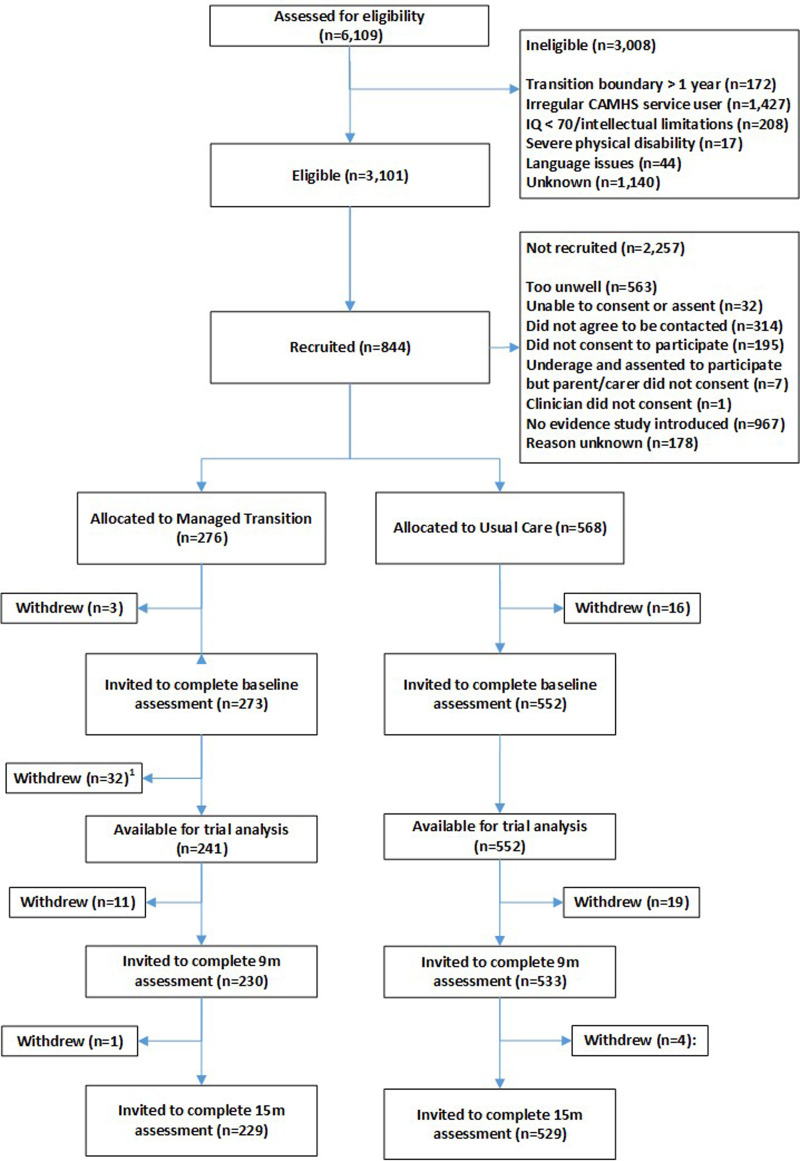
Participant flow through the trial.

Participant flow through the trial, including numbers screened, numbers recruited, withdrawals and loss to follow-up are shown in Fig. 1. In all, 844 YP were recruited (1st Oct 2015–31st Dec 2016) from 40 CAMHS, including 19 YP who withdrew before baseline assessment. A further 32 participants (at a single site in Croatia) were excluded owing to uncertainty concerning validity of participant consent, so 793 were available for the baseline assessments [Table 1 (abridged), online Supplementary Table S1 (full version)]; 273 in the MT group and 552 in the UC group. Attrition rates at 15 months were similar in the trial arms (4% UC group; 5% MT group). Reasons for withdrawal, where given, were similar in both trial arms – mainly being too busy and not wanting to talk about mental health problems. Online Supplementary Fig. S5 is a CONSORT diagram illustrating the number and sizes of clusters (CAMHS) recruited and randomised to the MILESTONE study (cRCT, economic evaluation and longitudinal cohort study). Further information about participating CAMHS (whether community and/or hospital based, existence of transition policy and means of funding) is given in online Supplementary Table S2. Baseline demographic and clinical characteristics of participants were generally well-balanced between the trial arms except that YP in the MT group were slightly more unwell (as shown by the HoNOSCA, TRAM and CGI scores, Table 1). Baseline characteristics of other participants (parent/carers, CAMHS and AMHS clinicians) are given by trial arm in online Supplementary Tables S5–S7.

**Table 1.** Baseline characteristics of participants (abridged version^a^)

	UC (*n* = 552)	MT (*n* = 241)	Total (*n* = 793)
Age (mean, s.d.)	17.48 (0.59)	17.64 (0.51)	17.53 (0.57)
Number (%) age unknown	5 (0.91%)	3 (1.25%)	8 (1.01%)
Gender (*N*, %)			
Female	340 (61.59%)	149 (61.83%)	489 (61.66%)
Male	211 (38.22%)	90 (37.34%)	301 (37.69%)
Prefer not to say	1 (0.18%)	2 (0.83%)	3 (0.38%)
Ethnicity (*N*, %)			
White	450 (81.52%)	210 (87.14%)	660 (83.23%)
Middle Eastern	1 (0.18%)	1 (0.41%)	2 (0.25%)
Asian	5 (0.91%)	7 (2.90%)	12 (1.51%)
Black/African/Caribbean	7 (1.27%)	4 (1.66%)	11 (1.39%)
Central or South American	6 (1.09%)	2 (0.83%)	8 (1.01%)
Mixed	12 (2.17%)	1 (0.41%)	13 (1.64%)
Unknown/Unspecified	71 (12.86%)	16 (6.64%)	87 (10.97%)
Country (*N*, %)			
Belgium	64 (11.57%)	33 (13.69%)	97 (12.23%)
Croatia	52 (9.42%)	0 (0.00%)^b^	52 (6.56%)
France	66 (11.93%)	13 (5.39%)	79 (9.96%)
Germany	64 (11.57%)	45 (18.67%)	109 (13.75%)
Ireland	12 (2.17%)	9 (3.73%)	21 (2.65%)
Italy	127 (22.97%)	63 (26.14%)	190 (23.96%)
Netherlands	75 (13.56%)	43 (17.84%)	118 (14.88%)
United Kingdom	92 (16.67%)	35 (14.52%)	127 (16.02%)
Primary clinical diagnosis (*N*, %)			
Substance-related and addictive disorders	3 (0.54%)	9 (3.73%)	12 (1.51%)
Schizophrenia spectrum and other psychotic disorders	26 (4.71%)	1 (0.41%)	27 (3.40%)
Depressive, bipolar and related disorders	104 (18.84%)	60 (24.90%)	164 (20.68%)
Anxiety disorders	69 (12.50%)	25 (10.37%)	94 (11.85%)
Obsessive-compulsive and related disorders	14 (2.54%)	4 (1.66%)	18 (2.27%)
Trauma- and stressor-related disorders	21 (3.80%)	24 (9.96%)	45 (5.67%)
Dissociative disorders	0 (0.00%)	1 (0.41%)	1 (0.13%)
Somatic symptoms and related disorders	9 (1.63%)	2 (0.83%)	11 (1.39%)
Feeding and eating disorders	45 (8.15%)	9 (3.73%)	54 (6.81%)
Disorders of adult personality and behaviour	24 (4.35%)	11 (4.56%)	35 (4.41%)
Gender dysphoria	4 (0.72%)	2 (0.83%)	6 (0.76%)
Other disorders of adult personality and behaviour	1 (0.18%)	0 (0.00%)	1 (0.13%)
Neurodevelopmental disorders^c^	186 (33.70%)	71 (29.46%)	257 (32.41%)
Other^d^	3 (0.54%)	1 (0.41%)	4 (0.50%)
Unspecified/other mental disorder	2 (0.36%)	2 (0.83%)	4 (0.50%)
Multiple primary diagnoses	21 (3.80%)	9 (3.73%)	30 (3.78%)
None	20 (3.62%)	10 (4.15%)	30 (3.78%)
Time spent under CAMHS care (*N*, %)			
Less than 6 months	52 (9.42%)	43 (17.84%)	95 (11.98%)
6 months to 1 year	90 (16.30%)	42 (17.43%)	132 (16.65%)
1 year to 2 years	91 (16.49%)	30 (12.45%)	121 (15.26%)
2 years to 5 years	145 (26.27%)	57 (23.65%)	202 (25.47%)
5 to 10 years	76 (13.77%)	37 (15.35%)	113 (14.25%)
More than 10 years	49 (8.88%)	17 (7.05%)	66 (8.32%)
Unknown	49 (8.88%)	15 (6.22%)	64 (8.07%)
HoNOSCA (mean, s.d.)	11.60 (6.86)	13.78 (7.11)	12.14 (6.98)
Number missing (%)	23 (4.17%)	12 (4.98%)	35 (4.41%)
HoNOSCA Self Report (SR) (mean, s.d.)	12.26 (9.23)	12.72 (8.33)	12.40 (8.96)
Number missing (%)	39 (7.07%)	15 (6.22%)	54 (6.81%)
TRAM (clinician report)			
Subscales (mean, s.d.)			
Symptoms	17.74 (9.97)	21.23 (9.95)	18.85 (10.09)
Risk	14.70 (12.50)	20.11 (13.06)	16.42 (12.92)
Overall disruption	24.89 (18.40)	26.28 (15.97)	25.33 (17.67)
Factors affecting symptoms	23.86 (18.59)	27.40 (20.26)	24.99 (19.20)
Barriers to functioning	26.12 (14.20)	27.77 (13.58)	26.64 (14.02)
Overall illness (takes into account all symptoms across all existing conditions) (*N*, %)			
Recovered – ongoing treatment not required	59 (10.69%)	24 (9.96%)	83 (10.47%)
Recovered – symptoms absent as long as on treatment	80 (14.49%)	22 (9.13%)	102 (12.86%)
Mildly ill	102 (18.48%)	45 (18.67%)	147 (18.54%)
Moderately ill	142 (25.72%)	84 (34.85%)	226 (28.50%)
Severely ill	77 (13.95%)	41 (17.01%)	118 (14.88%)
Very severely ill	7 (1.27%)	3 (1.24%)	10 (1.26%)
Unknown	85 (15.40%)	22 (9.13%)	107 (13.49%)
Number (%) YP with no TRAM CR at T1	85 (15.40%)	22 (9.13%)	107 (13.49%)
WHO Quality of Life Brief Inventory (WHOQOL-BREF) (mean, s.d.)			
Physical health	14.84 (2.66)	14.41 (2.93)	14.71 (2.75)
Psychological	12.20 (3.56)	11.83 (3.57)	12.09 (3.57)
Social relationships	13.72 (3.31)	13.99 (3.36)	13.80 (3.33)
Environment	15.15 (2.64)	14.86 (2.52)	15.06 (2.60)
Number (%) missing	48 (8.70%)	26 (10.79%)	74 (9.33%)
EQ-5D-5L (health status) (mean, s.d.)	0.78 (0.20)	0.78 (0.20)	0.78 (0.20)
Number (%) missing	47 (8.51)	25 (10.37)	72 (9.08)
Youth Self Report (YSR), Adult Self Report (ASR) (*t* scores) (mean, s.d.)			
Internalising problems	60.62 (12.56)	63.42 (12.73)	61.47 (12.67)
Externalising problems	51.54 (10.61)	53.64 (9.67)	52.18 (10.37)
Number (%) missing	55 (9.96)	24 (9.96)	79 (9.96)
Child Behaviour Checklist (CBCL), Adult Behaviour Checklist (ABCL) (*t* scores) (mean, s.d.)			
Internalising problems	63.62 (10.90)	65.81 (10.42)	64.24 (10.80)
Externalising problems	53.75 (10.23)	57.15 (10.67)	54.72 (10.47)
Number (%) missing	137 (24.82)	75 (31.12)	212 (26.73)
Severity of illness (CGI)			
Not assessed (0)	13 (2.36%)	5 (2.07%)	18 (2.27%)
Normal, not at all ill (1)	33 (5.98%)	12 (4.98%)	45 (5.67%)
Borderline mentally ill (2)	75 (13.59%)	30 (12.45%)	105 (13.24%)
Mildly ill (3)	114 (20.65%)	44 (18.26%)	158 (19.92%)
Moderately ill (4)	128 (23.19%)	70 (29.05%)	198 (24.97%)
Markedly ill (5)	73 (13.22%)	45 (18.67%)	118 (14.88%)
Severely ill (6)	34 (6.16%)	11 (4.56%)	45 (5.67%)
Among the most extremely ill patients (7)	6 (1.09%)	3 (1.24%)	9 (1.13%)
Unknown	76 (13.77%)	21 (8.71%)	97 (12.23%)

a For full baseline characteristics see online Supplementary Table S1.

b We randomised three clusters from Croatia (two to the UC arm and one to MT) but had to withdraw one of the clusters (MT arm) from the study due to uncertainty regarding the validity of participant consent. The data collected from this site are therefore excluded from the analysis.

c Includes 42 with specific learning disorders (36 UC, 6 MT), 74 with autism (56 UC, 18 MT) and 97 with ADHD (69 UC, 28 MT).

d Includes relational problems and other circumstances of personal history.

### Transition decisions

To inform transition decisions, 219 TRAM summary reports were produced for clinicians in the MT arm, relating to 91% of the YP in that group. At 15 months follow-up, 26.5% of participants were still under the care of their original CAMHS (27.4% UC *v.* 24.5% MT) (online Supplementary Table S8). Clinicians in the MT group recommended continuing in CAMHS slightly more frequently than in the UC group (25.0% UC *v.* 28.6% MT) and also transition to AMHS (21.0% UC *v.* 27.0% MT). Slightly more YP had been accepted by, and were under the care of, their new service at 15 months follow-up in the MT group compared to UC (22.1% UC *v.* 28.2% MT). The number of YP rejected by or not yet seen by the new service was small (0.54% UC *v.* 1.2% MT). In the MT group, 35.3% of clinicians reported sharing, or intending to share, the TRAM findings with the young person, despite the fact this was not a requirement of the intervention. Similarly, a very small number of CAMHS clinicians in the UC group (3.44%) also reported having shared, or intending to share, the TRAM findings with the young person but as they did not receive the TRAM summary report, these responses must (if not reporting errors) refer to the clinician version of the TRAM and may therefore indicate contamination.

### Primary outcome

Unadjusted mean HoNOSCA scores differed significantly between the MT and UC groups at baseline and at 15 months, and declined over time in both groups (online Supplementary Fig. S6). This indicated general improvement in mental health and wellbeing over time in both groups but the reduction appeared more rapid in the MT group (online Supplementary Fig. S7). The difference in mean HoNOSCA scores between the trial arms (MT-UC) at 15 months, estimated by the GLMM, was −1.11 [95% confidence interval (CI) −2.07 to −0.14, *p* = 0.03] (Table 2). The difference between the UC and MT trial arms in the rate of change in the HoNOSCA score over the study period (baseline to 15 months) was −1.70 (95% CI −2.97 to −0.43, *p* = 0.009). The intraclass-correlation coefficient between HoNOSCA scores in the same country was 0.004 (95% CI 0.000–0.782), between HoNOSCA scores in the same cluster and country was 0.037 (95% CI 0.018–0.076) and between HoNOSCA scores in the same participant, cluster and country was 0.110 (95% CI 0.068–0.173). Country, cluster and individual random-effects accounted for only 12.3% of the total residual variation. The results of sensitivity, subgroup and exploratory analyses of the primary outcome are presented in the online Supplementary appendix (Figs S8 and S9).

**Table 2.** Statistical analysis of primary and secondary outcomes 15 months after entry to the study

	*N* (%)	UC (mean score at 15 months) (*N* = 552)	*N* (%)	MT (mean score at 15 months) (*N* = 241)	Difference and 95% CI	*p* value
Primary outcome						
HoNOSCA	429 (77.72)	8.82 (6.25)	173 (71.78)	8.63 (6.00)	−1.11 (−2.07 to 0.14)^a^	0.03
Secondary outcomes						
HoNOSCA SR	393 (71.20)	10.72 (8.91)	174 (72.20)	9.52 (7.35)	−1.71 (−2.88 to 0.55)^b^	0.004
WHOQOL-BREF						
Physical health	371 (67.21)	50.20 (13.21)	158 (65.56)	48.62 (10.99)	−1.07 (−3.34 to 1.19)^c^	0.35
Psychological	371 (67.21)	51.22 (16.71)	158 (65.56)	51.03 (14.49)	0.91 (−2.15 to 3.97)^c^	0.56
Social relationships	371 (67.21)	62.92 (20.59)	158 (65.56)	62.03 (19.40)	1.28 (−2.76 to 5.32)^c^	0.54
Environment	371 (67.21)	67.92 (16.61)	158 (65.56)	67.25 (14.47)	1.27 (−1.83 to 4.36)^c^	0.42

a Mean difference in the HoNOSCA score between the trial arms (MT–UC) at 15 months, from a multilevel model with four levels (country, site, participant and follow-up time) and adjustment for gender, baseline HoNOSCA score and diagnosis (categorised as anxiety, depression, neurodevelopmental disorder or other).

b Mean difference in HoNOSCA-SR score between the trial arms (MT–UC) at 15 months, from a multilevel model with four levels (country, site, participant and follow-up time) and adjustment for gender, baseline HoNOSCA-SR score and diagnosis (categorised as anxiety, depression, neurodevelopmental disorder or other).

c Mean difference in WHOQOL-BREF score between the trial arms (MT–UC) at 15 months, from a multilevel model with four levels (country, site, participant and follow-up time) and adjustment for gender, baseline WHOQOL-BREF score and diagnosis (categorised as anxiety, depression, neurodevelopmental disorder or other).

### Secondary outcomes

After adjusting for HoNOSCA-SR at entry, gender, clinical diagnosis and clustering, the difference in mean HoNOSCA-SR scores between the trial arms (MT-UC) at 15 months was −1.71 (95% CI −2.88 to −0.55, *p* = 0.004) (Table 2). There was no significant difference in the quality of life ratings (WHO-BREF) between the trial groups at 15 months (after adjusting for clustering, baseline WHO-BREF score, gender and clinical diagnosis). Other secondary outcomes are summarised in Table 3. As these data have not been adjusted to account for clustering and imbalances in clinical and demographic factors at baseline, they should be interpreted cautiously. There were no marked differences between the UC and MT groups for the majority of measures [IBDCS, YSR/ASR, CBCL/ABCL, number of life events, CGIS, EQ-5D, SLOF, OYOF-TES (YP), OYOF-END (YP), OYOF-END (PC) and TROM] but parents/carers of YP who were to transition to AMHS did report satisfaction with end of care, and satisfaction with preparation for end of care, slightly more often in the MT group than with UC group (38.6% *v.* 35.5% and 40.2% *v.* 37.8%, respectively). There was no difference in overall satisfaction between these groups, however, with parent/carers in both groups reporting fairly high levels of satisfaction overall (average scores were 7/10 on a 0–10 scale, where 0 indicates completely unsatisfied and 10 fully satisfied).

**Table 3.** Summary of other secondary outcomes 15 months after entry to the study^a^

	*N* (%^b^)	UC	*N* (%^c^)	MT
Independent Behaviour During Consultation Scale (mean, s.d.)	196 (35.51)	15.98 (6.76)	83 (34.44)	15.48 (5.78)
Number (%) missing^d^	356 (64.49)		158 (65.56)	
Youth Self Report/Adult Self Report (YSR/ASR) (*t* scores)				
Internalising problems	381 (69.02)	60.23 (14.62)	161 (66.81)	60.08 (13.05)
Externalising problems	381 (69.02)	51.24 (11.00)	161 (66.81)	51.05 (9.55)
Number (%) missing	171 (30.98)		80 (33.19)	
Child Behaviour Checklist/Adult Behaviour Checklist (CBCL/ABCL) (*t* scores)				
Internalising problems	310 (56.16)	58.94 (11.65)	110 (45.64)	58.89 (11.65)
Externalising problems	310 (56.16)	52.35 (9.24)	110 (45.64)	52.90 (9.10)
Number (%) missing	242 (43.84)		131 (54.36)	
Number of life events [median (IQR)]	385 (69.75)	1 (0–2)	164 (68.05)	1 (0–2)
Number (%) missing	167 (30.25)		77 (31.95)	
Severity of illness (CGIS)				
Not assessed (0)	8 (1.45)		0 (0.00)	
Normal, not at all ill (1)	9 (1.63)		7 (2.90)	
Borderline mentally ill (2)	19 (3.44)		11 (4.56)	
Mildly ill (3)	37 (6.70)		16 (6.64)	
Moderately ill (4)	43 (7.79)		18 (7.47)	
Markedly ill (5)	16 (2.90)		13 (5.39)	
Severely ill (6)	11 (1.99)		4 (1.66)	
Among the most extremely ill patients (7)	1 (0.18)		0 (0.00)	
Unknown^e^	408 (73.91)		172 (71.37)	
EQ-5D (mean, s.d.)	390 (70.65)	0.79 (0.21)	168 (69.71)	0.80 (0.21)
Number (%) missing	162 (29.35)		73 (30.29)	
Specific Levels of Functioning Scale (SLOF) [median (IQR)]				
Physical functioning	311 (56.34)	25 (24–25)	113 (46.89)	25 (24–25)
Personal care skills	311 (56.34)	35 (33–35)	113 (46.89)	35 (33–35)
Interpersonal relationships	311 (56.34)	28 (22–33)	113 (46.89)	27 (22–33)
Social acceptability	311 (56.34)	34 (31–35)	113 (46.89)	33 (32–35)
Activities	311 (56.34)	53 (49–55)	113 (46.89)	53 (49–55)
Work skills	311 (56.34)	26 (21–29)	113 (46.89)	24 (20–30)
Number (%) missing	241 (43.66)		128 (53.11)	
On Your Own Feet – Transition Experience Scale (OYOF-TES)^f^ (young person) (mean, s.d.)				
Satisfaction with end of care	126 (22.83)	36.93 (8.57)	69 (28.63)	36.32 (8.64)
Preparation for end of care	126 (22.83)	32.67 (7.84)	69 (28.63)	31.90 (8.11)
Overall satisfaction (scale 0–10)	126 (22.83)	6.14 (2.85)	69 (28.63)	6.12 (2.54)
OYOF-END^g^ (end of care) (young person) (mean, s.d.)				
Satisfaction with end of care	202 (36.59)	18.35 (4.00)	82 (34.02)	19.29 (3.48)
Preparation for end of care	202 (36.59)	29.38 (7.46)	82 (34.02)	30.59 (7.37)
Overall satisfaction (scale 0–10)	202 (36.59)	6.98 (2.54)	82 (34.02)	6.95 (2.50)
OYOF-TES^f^ (parent/carer) (mean, s.d.)				
Satisfaction with end of care	97 (17.57)	35.53 (9.32)	58 (24.07)	38.57 (7.18)
Preparation for end of care	97 (17.57)	37.82 (10.77)	58 (24.07)	40.16 (8.40)
Overall satisfaction (scale 0–10)	97 (17.57)	6.14 (2.73)	58 (24.07)	6.52 (2.48)
OYOF-END^g^ (parent/carer) (mean, s.d.)				
Satisfaction with end of care	169 (30.62)	17.66 (5.09)	53 (21.99)	18.66 (3.75)
Preparation for end of care	169 (30.62)	27.23 (7.98)	53 (21.99)	28.13 (7.14)
Overall satisfaction (scale 0–10)	169 (30.62)	6.99 (2.78)	53 (21.99)	7.19 (2.47)
Transition related outcomes^h^ (TROM) (clinician report)				
Overall severity since transition	14 (2.54)		5 (2.07)	
Recovered – no treatment required	48 (8.70)		16 (6.64)	
Recovered – treatment required	87 (15.76)		27 (11.20)	
Mildly ill	114 (20.65)		40 (16.60)	
Moderately ill	35 (6.34)		19 (7.88)	
Severely ill	8 (1.45)		2 (0.83)	
Very severely ill	246 (44.57)		109 (45.23)	
Unknown	14 (2.54)		5 (2.07)	

a As there was no adjustment for the hierarchical nature of the data or imbalances in clinical and demographic factors at baseline, the descriptive data should be interpreted cautiously.

b Calculated as the proportion of the UC group (*N* = 552).

c Calculated as the proportion of the MT group (*N* = 241).

d Completed only if the young person is still under the care of CAMHS/AMHS at 15 months.

e CGIS is only measured in those who are service users – hence missing reflects Not Applicable as well as true missing.

f Completed if the transition decision was to refer the young person to AMHS.

g Completed if the transition decision was to discharge the young person from mental health services.

h The TROM was completed at the first follow-up after the transition decision had been made, at either of the 9 and 15 months follow-up.

Self-harm (suicide, suicide attempt and suicidal thoughts) was the most common serious adverse event (online Supplementary Table S9). None were assessed to be related to participation or the intervention, and there was no difference in pattern, severity or frequency of SAEs between trial arms.

The intervention was relatively inexpensive to implement, with direct intervention delivery costs ranging between €17 and €65 per child and clinician training costs ranging between €22 and €176, depending on how training and delivery was conducted in each country.

## Discussion

This is the first-ever RCT of a scalable intervention for improving mental health outcomes for YP at the child–adult SB. We met our original recruitment target and retention of participants was better than expected: 15-month primary outcome was assessed in 76% (602/793) of the cohort across eight countries. Overall, the trial covered 38 child/adolescent services in eight European countries making the study findings generalisable to a range of settings.

We developed and tested a new intervention, MT, in eight European countries (Singh et al., 2017). Our model, based on available evidence (Paul, Street, Wheeler, & Singh, 2015), includes structured assessments of transition need, readiness and appropriateness, and facilitated shared decision-making between YP, parent/carer, CAMHS clinician and, where appropriate, with AMHS clinicians. The intervention uses relatively few resources, is easily incorporated into routine clinical practice and is generalisable to scale. We found that compared to UC, MT led to a small improvement in overall mental health and wellbeing of YP 15 months after entry to the trial. Furthermore, improvement was more rapid than with UC.

Our MT model ensures that the process of transition is planned and purposeful, and addresses the needs of YP as they reach the upper age SB and move towards independence (Paul et al., 2013). In our model, young person and their carer are closely involved in preparation for leaving one service and joining another, with adequate information sharing and a service alignment to maintain therapeutic continuity (Cleverley et al., 2020; Singh et al., 2016). Our model of MT is relatively easy to implement: data collation can be fully automated within a web-based platform and incorporated into routine care. The paper-version of the TRAM is available in seven languages and is free of charge to charities and publicly funded organisations (requests to corresponding author). Although the need for such a model has often ben articulated, our study shows that transition process can be improved with modest investment of time and resources. TRAM and the MT model may also lend themselves, with appropriate modifications, to other clinical settings and disciplines where transition between services is unsatisfactory (Hart, Patel-Nguyen, Merkley, & Jonas, 2019).

Previous attempts at improving service transition in mental health are few, and no RCT has ever been conducted (Appleton et al., 2019; Paul et al., 2015). A recent study used a ‘shared management model’ with individualised transitional care plans and a transition coordinator (Cappelli et al., 2016) and another a streamlined transition process for YP in an attention deficit hyperactivity disorder transition clinic (Moosa & Sandhu, 2015). Both reduced the number of YP waiting for a referral and improved access to AMHS, but did not measure mental health outcomes after transition. Another recent innovation is extending the CAMHS boundary beyond 18, such as in Australia (McGorry, Bates, & Birchwood, 2013) and in the UK (Norfolk and Birmingham). In Norfolk, UK, a redesigned 14–25 year service increased referrals to the service, yet the proportion of accepted referrals dropped (Maxwell et al., 2019). The 14–25 model also risks creating another SB at 14. An evaluation of the 0–25 service in Birmingham, UK, found that a shortage of medical staff, poor service infrastructure, inadequate or incompatible data management systems, among other things, hampered care provision despite widespread support for the model (Birchwood et al., 2018).

Our MT model provides a solution that can be applied within existing service structures. For more robust transitional health care for YP, the MT model could be implemented as part of a comprehensive package of service alignment. The MT model is far less resource intensive than large-scale service reorganisation such as the 0–25 model, multi-component transition-specific programmes, or youth-friendly service models (Hetrick et al., 2017; McGorry et al., 2013). The overall service alignment should take other aspects of transition into account (Cleverley et al., 2020; Singh et al., 2016), including transition to non-mental health services (Appleton et al., 2019).

The modest clinical gains of our trial need to be interpreted in the context of the need for a single quantitative primary outcome measure for a process-related study. Finding a suitable measure for the primary outcome was difficult, owing to the nature of the intervention and clinical diversity of the participants. We decided on HoNOSCA because it was developed for child/adolescent mental health care settings and is used widely in Europe (Garralda et al., 2000; Harris et al., 2018). Baseline HoNOSCA scores were low in the trial (mean = 12.14, s.d. = 6.98), in keeping with comparative groups in the UK (mean = 13.71) (Gowers et al., 1999) and Italy (mean = 13.6) (D'Avanzo et al., 2018). The reduction in the mean HoNOSCA score observed with MT (1.11 points) therefore corresponds to an approximate 9% relative reduction in the HoNOSCA score, equivalent to each participant having a 1 point reduction in symptom severity on 1–2 of the 13 questions (from moderately severe to mild, say, or from severe to moderately severe).

We were surprised at the relatively large proportion of YP still in CAMHS or without a transition decision at 15 months, but similar findings have been confirmed in a recent systematic review (Appleton et al., 2019). It perhaps reflects the diverse funding structures across the EU, with CAMHS in some countries able and willing to continue providing care beyond the SB (Signorini et al., 2017, 2018). Discharge of YP who do not require transition to AMHS may be challenging for CAMHS clinicians because many primary care services feel under-resourced and poorly equipped to meet the complex needs of these YP (Tatlow-Golden, Prihodova, Gavin, Cullen, & McNicholas, 2016).

There are, as in any trial, limitations of this study. The most unwell may be underrepresented; so we may have missed some for whom transition is essential but particularly challenging. We could not blind the CAMHS clinicians or assessors; the former was unavoidable given the nature of the intervention, but the latter was imposed due to resourcing issues. Blinding of assessors would have required two research assistants per recruitment site: one to deal with training, recruitment and the administrative aspects of the trial and another to undertake HoNOSCA interviews and assessments. This may also have made it more difficult for our research assistants to bond with participants, as each participant would now have two contacts within the research team to develop a relationship with rather than one. For clinicians in the UC arm, knowledge of the trial may have enhanced their focus on transition options. Furthermore, although, clinicians in the UC arm did not receive the TRAM summary report they did complete the clinician version of the TRAM, which may also have influenced their decision making.

## Data Availability

Requests for original (fully anonymised) participant data may be made to the corresponding author.

## Supplementary material

For supplementary material accompanying this paper visit https://doi.org/10.1017/S0033291721003901. click here to view supplementary material
