# Supplementary material for: Effect of managed transition on mental health outcomes for young people at the child–adult mental health service boundary: a randomised clinical trial
Source: Psychol Med. 2021 Oct 29;53(6):2193–204. doi: 10.1017/S0033291721003901 (PMC10123823; doi:10.1017/S0033291721003901)
Supplement: Supplementary file 1 [file S0033291721003901sup001.docx]

**Effect of Managed Transition on mental health outcomes for young people at the child-adult mental health service boundary: a randomised clinical trial - Supplementary Appendix**

This supplement consists 29 pages.

Table of Contents

[Trial oversight and patient and public involvement 2](#_Toc69142991)

[Data Collection 2](#_Toc69142992)

[Further Statistical and Health Economic Methods 2](#_Toc69142993)

[Randomisation 2](#_Toc69142994)

[Statistical Analyses 2](#_Toc69142995)

[Efficacy analyses 2](#_Toc69142996)

[Calculation of intra-cluster correlation coefficient (ICC) 2](#_Toc69142997)

[Sensitivity analyses assessing the impact of missing data 3](#_Toc69142998)

[Subgroup analyses 3](#_Toc69142999)

[Intervention costing 3](#_Toc69143000)

[Results of sensitivity, subgroup and exploratory analyses 3](#_Toc69143001)

[References 4](#_Toc69143002)

[MILESTONE Consortium 5](#_Toc69143003)

[Figure S1: Logic model of managed transition 7](#_Toc69143004)

[Figure S2: Example of TRAM summary report 8](#_Toc69143005)

[Figure S3: Email to clinicians that accompanied the TRAM summary report 9](#_Toc69143006)

[Figure S4: Transition leaflet that accompanied the TRAM summary report 10](#_Toc69143007)

[Figure S5: Recruitment and allocation of clusters 12](#_Toc69143008)

[Figure S6: Mean observed HoNOSCA score and standard errors at baseline, 9 months and 15 months 13](#_Toc69143009)

[Figure S7: Change in observed mean HoNOSCA score since entry to the study 14](#_Toc69143010)

[Figure S8: Primary outcome (HoNOSCA score) by pre-planned subgroups 15](#_Toc69143011)

[Figure S9: Exploratory analysis of the difference between the trial arms by HoNOSCA domain 16](#_Toc69143012)

[Table S1: Baseline characteristics of participants (full version) 17](#_Toc69143013)

[Table S2: Characteristics of participating CAMHS (clusters) 21](#_Toc69143014)

[Table S3: Schedule of data collection 22](#_Toc69143015)

[Table S4: List of scales used and their purpose 23](#_Toc69143016)

[Table S5: Baseline characteristics of parent/carers 24](#_Toc69143017)

[Table S6: Characteristics of CAMHS clinicians 25](#_Toc69143018)

[Table S7: Characteristics of AMHS clinicians 26](#_Toc69143019)

[Table S8: Transition decisions and referrals by CAMHS clinicians 27](#_Toc69143020)

[Table S9: Number, nature, severity and relatedness of severe adverse events (SAE) in each treatment group 28](#_Toc69143021)

[Table S10: Intervention costing example – UK 29](#_Toc69143022)

## Trial oversight and patient and public involvement

An external scientific, ethical, and clinical advisory board (SCEAB) provided independent oversight. Patient and public involvement (PPI), comprising ten young project advisors (with recent experience of transition) and a group of parents/carers from England and Ireland contributed to the methodology, design, refinement of tools, data collection, and interpretation of results.

## Data Collection

Assessments (HoNOSCA interviews) could be deferred by up to three months if a young person was too unwell to take part within the allotted time frame. Assessments at 9 and 15 months were intended to be completed remotely (telephone and online) to save on resources, however, a face-to-face meeting was arranged at a convenient location, if this was feasible and preferred by the young person.

## Further Statistical and Health Economic Methods

### Randomisation

Unequal randomisation was chosen for the cRCT to reduce the number of intervention sites (allowing better use of resources) and to ensure sufficient numbers of control participants would be available for the associated longitudinal cohort study. Randomisation was performed on a country by country basis by the trial statistician, using a computer- generated randomisation sequence, prior to the study opening in each country. Firstly, for countries where the number of eligible clusters exceeded three, CAMHS were randomly selected in groups of three for participation in the cRCT and any remainder allocated to the associated longitudinal cohort study. Secondly, CAMHS selected for inclusion in the cRCT were randomised 2:1 between UC and MT. Clusters recruited after randomisation took place were not included in the cRCT but allocated directly to the associated longitudinal study.

### Statistical Analyses

Continuous variables that follow an approximately Normal distribution were summarised by their mean and standard deviation. Variables that appeared to deviate significantly from normality were summarised using the median and interquartile range. Categorical data were presented as frequencies and percentages.

### Efficacy analyses

The 15-month follow up was chosen for the primary outcome because it was anticipated that the majority would have moved on from CAMHS by then, given that to enter the study they had to be approaching the CAMHS boundary. Multilevel models employed a four-level random effects hierarchy (to allow assessments nested to participants nested to clusters nested to countries). HoNOSCA score at month 15 was the dependent variable and treatment group an independent variable. Adjustment was also made in the models for baseline HoNOSCA score, time-point, gender and diagnosis (fixed-main effects) and time by treatment, gender by treatment and time by baseline score (fixed-interaction effects). We used only random intercepts (RIM) as slopes (RISM) did not benefit our model (based on BIC results). In addition, we allowed residuals to have their own correlation structure to adjust for some heteroskedastic errors. Owing to small numbers, diagnoses were grouped into five categories (anxiety, depression, neurodevelopmental disorders, eating disorders, other) for inclusion in the model. Model assumptions were assessed by examining influential values (outliers) and diagnostic plots of residuals. Normality of the random effects and homoscedasticity of errors were assessed graphically. Alternative correlation structures were explored and the model with the lowest information criterion (BIC) selected. Analysis of the primary outcome was undertaken using linear mixed model. In the fixed-effect part, HoNOSCA score was modelled as a function of trial arm (usual care vs. managed transition) with adjustment for gender, HoNOSCA score at baseline, diagnosis at baseline and appropriate interactions (trial arm x time, trial arm x gender and trial arm x HoNOSCA score at baseline). For the random part, the levels used were patient (level 1), cluster (level 2) and country (level 3), as justified by likelihood ratio tests and the magnitude of the intra-cluster correlation coefficient. Models with random intercepts only were found to be sufficient. Variance of errors differed over time and between arms. We accommodated this by allowing for Toeplitz correlation at the intercepts level^1^. There were no problems with convergence. The difference in the width of confidence intervals between homoscedastic and heteroscedastic treatment effect was about 15%.

### Calculation of intra-cluster correlation coefficient (ICC)

The intra-cluster correlation coefficient (ICC) of the primary outcome (proportion of the variance due to between-cluster variation) was estimated from the model using the following formula:

$$\rho={\sigma_{b}}^{2}/({\sigma_{b}}^{2}+{\sigma_{w}}^{2})$$

where ${\sigma_{w}}^{2}$ is the within-cluster variance, which is assumed to be the same in all clusters, and ${\sigma_{b}}^{2}$ is the between-cluster variance. ${\sigma_{b}}^{2}$and ${\sigma_{w}}^{2}$ can be estimated from the mixed effects model. The cluster-specific model is $Y_{ij}= \alpha+\beta x_{ij}+\mu_{i}+\varepsilon_{ij}$ where$\mu_{i}\sim N(0{,\sigma_{b}}^{2})$, the residuals$\varepsilon_{i}\sim N(0, {\sigma_{w}}^{2})$ and i denotes clusters and j denotes individuals. The ICC can therefore be estimated from the mixed effects model.

### Sensitivity analyses assessing the impact of missing data

If 10% or more of the values for the primary outcome were missing, missing values (whole scores) were imputed and the analysis of the primary outcome repeated. Imputation was only used if less than 40% of individual items in the scale were missing. The extent to which the assumptions of missing at random (MAR) and missing not at random (MNAR) were likely to hold were explored using Little’s test to measure the association between missingness and characteristics of YP at baseline and 15 months. These included country, trial arm, age, sex, education level, severity of illness (CGI), and whether the parent/carer was participating (as a proxy for parental engagement). Imputation was performed using chained equations assuming the multivariate normal distribution. Eight datasets were imputed for the missing data. The stability and appropriateness of any MI models were assessed and adjustments made to ensure convergence and that imputed values lay within plausible ranges. The same model was used to impute missing HoNOSCA at baseline and 15 months.

### Subgroup analyses

Pre-planned subgroup analyses of the primary outcome were diagnosis (depressive/ bipolar and related disorders/ anxiety disorders/ trauma and stressor related disorders/ feeding and eating disorders/ neurodevelopmental disorders/ other (including multiple primary)/ unknown), gender (male/female), ethnicity (white/not white), parental participation in the study (yes/no), hospitalisation (inpatient stay during study period) (yes/no), medication use during study period (yes/no), socioeconomic status (education level of parents), and illness severity at 15 months follow up (CGIS category). The study was not powered to detect differences between sub-groups. Any observed patterns should be interpreted extremely cautiously, owing to the smaller numbers and increased chance of Type I error.

*Exploratory analysis of the difference between the trial arms by HoNOSCA domain*

Analysis of the primary outcome was repeated for each HoNOSCA domain (behaviour, impairment, symptoms, social) separately. The study was not powered to detect differences between domains. Any observed patterns should be interpreted extremely cautiously, owing to the smaller numbers and increased chance of Type I error.

### Intervention costing

Data on staff time and other resources in the transition process were obtained at each location and costed using location-specific unit cost data where possible. This included PSSRU unit costs for the UK [1], Zorginstituut Nederland unit costs for the Netherlands [2], published unit costs for Italy [3], Belgium [4], France [4], and purchasing parity derived unit costs for Ireland (derived from the UK) as suggested by the Irish Health Information and Quality Authority guidelines for economic evaluation [5]. Unit costs for Germany were obtained from direct correspondence with the authors of the COFI study [6]. Training costs per patient were calculated by capturing the average time spent training intervention clinicians. This was multiplied by the number of intervention clinicians within each country and their unit costs, and then divided by the number of intervention patients to calculate a cost per patient. Given the high ratio of clinicians trained to intervention YPs, this is a very conservative cost estimate for training. For the delivery of the TRAM intervention, clinicians recorded the time spent completing and feeding back TRAM questionnaires and the trial team recorded the time taken to generate the TRAM reports. These were combined with unit costs to estimate TRAM costs. This resulted in location-specific estimates of the direct costs involved in implementing the managed transition process. Costs are presented in Belgian Euros using purchasing power parity for adjustment for the price year 2015. An example for the UK is presented in Table S10.

## Results of sensitivity, subgroup and exploratory analyses

Analysis of the primary outcome was repeated with imputation of missing items within the HoNOSCA scale (but not whole scores) using three different methods (multivariate normal regression, predictive mean matching and ordered logistic regression). The mean difference in HoNOSCA score between the trials arms using multivariate normal regression was -1.05 (95%CI -2.0 to -0.10), using predictive mean matching was -1.23 (95%CI -2.23 to -0.24) and using ordinal logistic regression -1.25 (95%CI -2.25 to -0.25). Owing to the pattern of missingness (when whole scores were missing so too were baseline covariates) it was not possible to impute whole scores.

Estimates of mean HoNOSCA scores and associated 95% confidence intervals for each subgroup separately are presented graphically in a forest plot (Figure S8). It appears that Managed Transition was most effective in those with other diagnoses (mean difference between trial arms =-2.09; 95%CI -4.01 to -0.17), those least unwell (CGIS ≤3) (mean difference between trial arms =-1.64; 95%CI -2.99 to -0.29) and those not on prescription medication (mean difference between trial arms =-1.53; 95%CI -2.97 to -0.10).

The primary efficacy analysis was repeated for each HoNOSCA domain separately. Estimates of the mean difference between the trials arms for each domain, together with 95% confidence interval, are presented in a forest plot in Figure S9. HoNOSCA symptom scores were lower in the Managed Transition group symptoms between (mean difference = -0.38; 95%CI -0.72 to -0.04) but there was no difference for the other domains.

###

## References

[1] ‘Unit Costs of Health and Social Care 2017 | PSSRU’. https://www.pssru.ac.uk/project-pages/unit-costs/unit-costs-2017/ (accessed Oct. 14, 2019).

[2] T. A. Kanters, C. A. M. Bouwmans, N. van der Linden, S. S. Tan, and L. Hakkaart-van Roijen, ‘Update of the Dutch manual for costing studies in health care’, *PLoS ONE*, vol. 12, no. 11, Nov. 2017, doi: 10.1371/journal.pone.0187477.

[3] A. Patel *et al.*, ‘Cost-effectiveness of adherence therapy versus health education for people with schizophrenia: randomised controlled trial in four European countries’, *Cost Eff. Resour. Alloc.*, vol. 11, no. 1, p. 12, May 2013, doi: 10.1186/1478-7547-11-12.

[4] G. Jerusalem *et al.*, ‘Patterns of resource utilization and cost for postmenopausal women with hormone-receptor–positive, human epidermal growth factor receptor-2–negative advanced breast cancer in Europe’, *BMC Cancer*, vol. 15, Oct. 2015, doi: 10.1186/s12885-015-1762-3.

[5] Health information and Quality Authority, ‘Guidelines for the Economic Evaluation of Health Technologies in Ireland’, 2019. [Online]. Available: https://www.hiqa.ie/sites/default/files/2019-07/HTA-Economic-Guidelines-2019.pdf.

[6] ‘Final Report Summary - COFI (Comparing policy framework, structure, effectiveness and cost-effectiveness of functional and integrated systems of mental health care) | Report Summary | COFI | FP7 | CORDIS | European Commission’. https://cordis.europa.eu/project/id/602645/reporting.

## MILESTONE Consortium

**MILESTONE Core members**: UK-West Midlands: Swaran Singh, Helena Tuomainen, Jason Madan, Moli Paul, Cathy Street, Dieter Wolke, Jane Warwick, Claire Daffern (University of Warwick); UK-London: Paramala Santosh, Federico Fiori (HealthTracker Ltd); Italy: Giovanni de Girolamo (Unit of Epidemiological and Evaluation Psychiatry - UOPEV, IRCCS Istituto Centro San Giovanni di Dio Fatebenefratelli, Brescia); France: Diane Purper-Ouakil (Centre Hospitalier Universitaire de Montpellier); Netherlands: Athanasios Maras (Yulius Academy), Frank Verhulst, Gwen C Dieleman, (Erasmus Medical Centre); Germany: Ulrike Schulze (University of Ulm); Andrea Wohner (concentris Research Management GmbH); Belgium: Sabine Tremmery (Katholieke Universiteit Leuven, Belgium); Republic of Ireland: Fiona McNicholas (University College Dublin, Republic of Ireland); Croatia: Tomislav Franić (University Hospital Split).

**Trial scientific, ethical, and clinical advisory committee:** Prof Maryann Davis, Prof Pat McGorry, Dr Adriana Mihai, and Prof Norman Sartorius

**MILESTONE research assistants: UK:** Priya Tah, Alastair Canaway, James Griffin, Rebecca Appleton, Philip Wells (University of Warwick), Natalie Heaney, Kate Lievesley, Mathilde Mastroianni, Jatinder Singh (Kings College London), Laura Adams (University of Plymouth); **Italy:** Giulia Signorini, Alessandro Ferrari, Elisa Gheza, Cecilia Ferrari, Laura Rivolta, Flavia Levi, Maria Cataldo, Lidia Manenti, Giorgia Morini, Adriana Pastore, Pamela Stagni, Cecilia Toselli, Pamela Varvara (Unit of Epidemiological and Evaluation Psychiatry - UOPEV, IRCCS Istituto Centro San Giovanni di Dio Fatebenefratelli, Brescia); France: Frédérick Russet, Virginie Maurice, Véronique Humbertclaude (Centre Hospitalier Universitaire de Montpellier); **Netherlands:** Larissa S van Bodegom, Mathilde M Overbeek (Yulius Academy); Suzanne E Gerritsen (Erasmus Medical Centre); **Germany:** Melanie Saam, Ulrike Breuninger (University of Ulm); **Belgium:** Gaëlle Hendrickx, Veronique De Roeck (Katholieke Universiteit Leuven); **Ireland:** Ingrid Holme, Aleksandra Gronostaj, Rachael McKenna, Lesley O’Hara (University College Dublin); **Croatia:** Nikolina Davidović, Helena Tomljenovic (University Hospital Split)

**MILESTONE young project advisors:** Amanda Tuffrey, Anna Wilson, Charlotte Gatherer, Leanne Walker (University of Warwick)

**MILESTONE recruitment sites, lead clinicians and staff:** **UK:** Jo Berriman (South Staffordshire and Shropshire Healthcare NHS Foundation Trust, UK), Vinuthna Pemmaraju (Black Country Partnership Foundation NHS Trust), Michael Slowik (Dudley & Walsall Mental Health Partnership NHS Trust), Ben Rogers, Alan Farmer (Worcestershire Health and Care NHS Trust), Paramala Santosh (South London and Maudsley NHS Foundation Trust); **Italy:** Giovanni Allibrio (Child and Adolescent Neuropsychiatry Unit, ASST Spedali Civili, Brescia), Marco Armandi (Child and Adolescent Neuropsychiatric Unit, Department of Neuroscience, IRCCS Bambino Gesù Children's Hospital, Rome; Department of Psychiatry, University of Geneva, Switzerland), Angelo Bertani (Department of Mental Health and Dependence, ASST Santi Paolo e Carlo, Milano), Giuseppe Carrà (Department of Mental Health, ASST Nord Milano, University of Milan Bicocca), Patrizia Conti (Child and Adolescent Neuropsychiatry Unit, ASST Lariana), Sabrina Ferrari (Child and Adolescent Neuropsychiatry Unit, AUSL of Parma, Parma), Francesco Margari (Psychiatric Clinic, Department of Basic Medical Sciences, Neuroscience and Sensory Organs, University of Bari ʺAldo Moroʺ, Bari) Ottaviano Martinelli (Child and Adolescent Neuropsychiatry Unit, ASST Lecco), Renata Nacinovich (Child and Adolescent Neuropsychiatry Unit, ASST Monza), Paolo Scocco (Department of Mental Health, ULSS 16 Padua, Italy; SOPROXI Onlus, Padua), Paolo Stagi (Child and Adolescent Neuropsychiatry Unit, AUSL OF Modena, Italy), Fabio Vanni (Programme on Adolescence, Department of Mental Health and Pathological Addiction, AUSL of Parma, Parma), Stefano Vicari (Child and Adolescent Neuropsychiatric Unit, Department of Neuroscience, IRCCS Bambino Gesù Children's Hospital, Rome); **France:** : Mario Speranza, Hélène Lida-Pulik (Centre Hospitalier de Versailles; INSERM CESP U 10 18 Université de Saclay), Renaud Jardri , Aesa Parenti (Université de Lille), David Da Fonseca, Isabelle Charvin (Centre Hospitalier Universitaire de Marseille), Frédérique Bonnet-Brilhault, Chrystèle Bodier, Catherine Prigent (Centre Hospitalier Universitaire de Tours), Aurélie Schandrin (Centre Hospitalier Universitaire de Nîmes); **Netherlands:** Therese van Amelsvoort (Department of Psychiatry and Psychology, Maastricht University, Maastricht), Albert Boon (Lucertis Child and Adolescent Psychiatry, Parnassia Psychiatric Institute, Rotterdam;Department of Child and Adolescent Psychiatry, Leiden University Medical Center/Curium, Leiden), Arno van Dam (GGZ Westelijk Noord-Brabant, Institute for Mental Health Care, Roosendaal); **Germany:** Jörg M. Fegert (University of Ulm), Renate Schepker, Elena Tanase, Beata Williams, (ZfP Südwürttemberg, Abteilung für Psychiatrie und Psychotherapie des Kindes-und Jugendalters Weissenau, Ravensburg) Michele Noterdaeme, Anne Sartor, Vehbi Sakar, Caroline von Bentzel (Josefinum Augsburg, Klinik für Kinder- und Jugenspsychiatrie und Psychotherapie, Augsburg); Belgium: Emmanuel Nelis (AZ Sint-Lucas Brugge), Dirk van West (ZNA Universitaire Kinder- en Jeugdpsychiatrie Antwerpen​), Catherine Klockaerts (KPC Genk), Ann Pepermans (CGG groep LITP), Marina Danckaerts (UPC KU Leuven), Anne Roekens (CGG VBO), Hanne Van Gutschoven (CGG Brussel), Christel Lippens (CGG Passant), Jean-Pierre Ermans (Rivage den Zaet); **Ireland:** Helen Keeley (CAMHS North Cork), Keith Holmes, James McDonald, Maria Migone, Fionnuala Lynch, Elaine Healy (Lucena), Peter Dineen (CAMHS, North Lee, Cork); **Croatia:** Vlatka Kovač, Katarina Dodig-Ćurković (University of Osijek, Osijek)

##
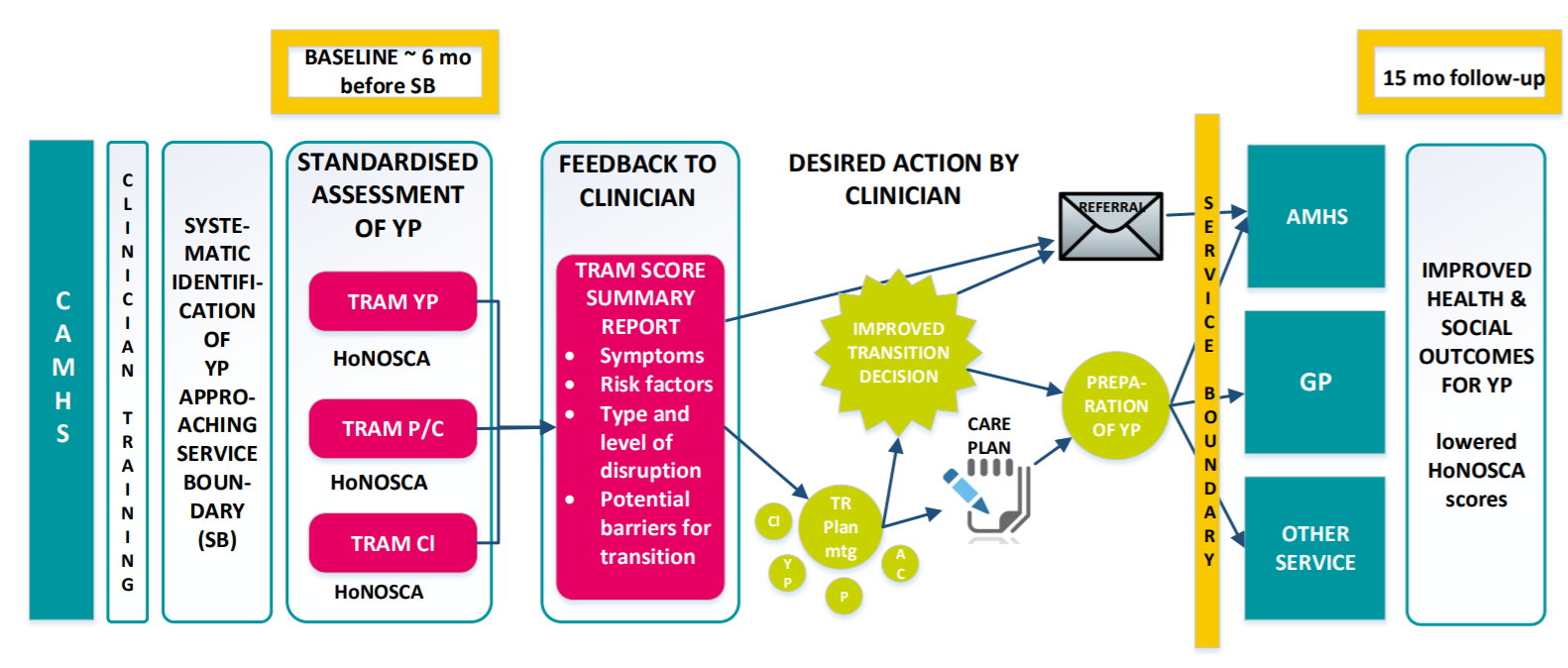
Figure S1: Logic model of managed transition

Managed Transition in its entirety comprises: 1) training of clinicians regarding optimal transition and TRAM; 2) a systematic identification of young people approaching the CAMHS service boundary; 3) the assessment of the young person using the Transition Readiness and Appropriateness Measure (TRAM) at approximately 6 months before the service boundary, with young person (YP), parent/carer (P/C) and clinician (Cl) completing bespoke versions of the TRAM; and 4) providing feedback to the clinician regarding TRAM scores from the three sources in a report; the report is attached to an email which explains the report and what steps clinicians can take to inform the transition decision (e.g., adding items with high score to a transition plan and discussing items with young person and parent/carer). Following this, it is the clinician’s decision whether to call a transition planning meeting, produce a care plan for transition, and prepare the young person for transition before the service boundary, should the young person need transition to adult services (AMHS). The TRAM report should be attached to the referral letter if further care in adult services is deemed necessary.

## Figure S2: Example of TRAM summary report

| 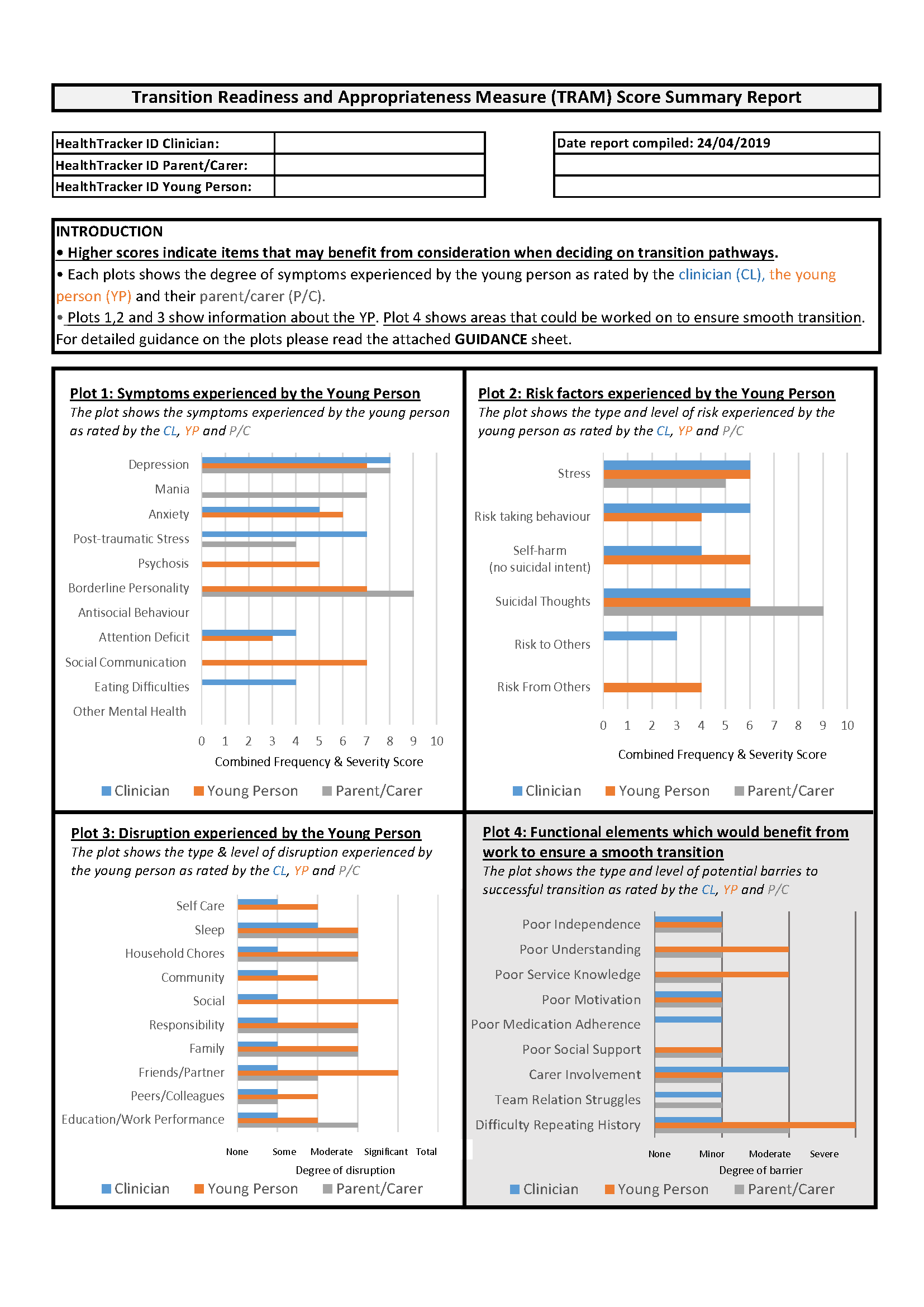 |
| --- |
| 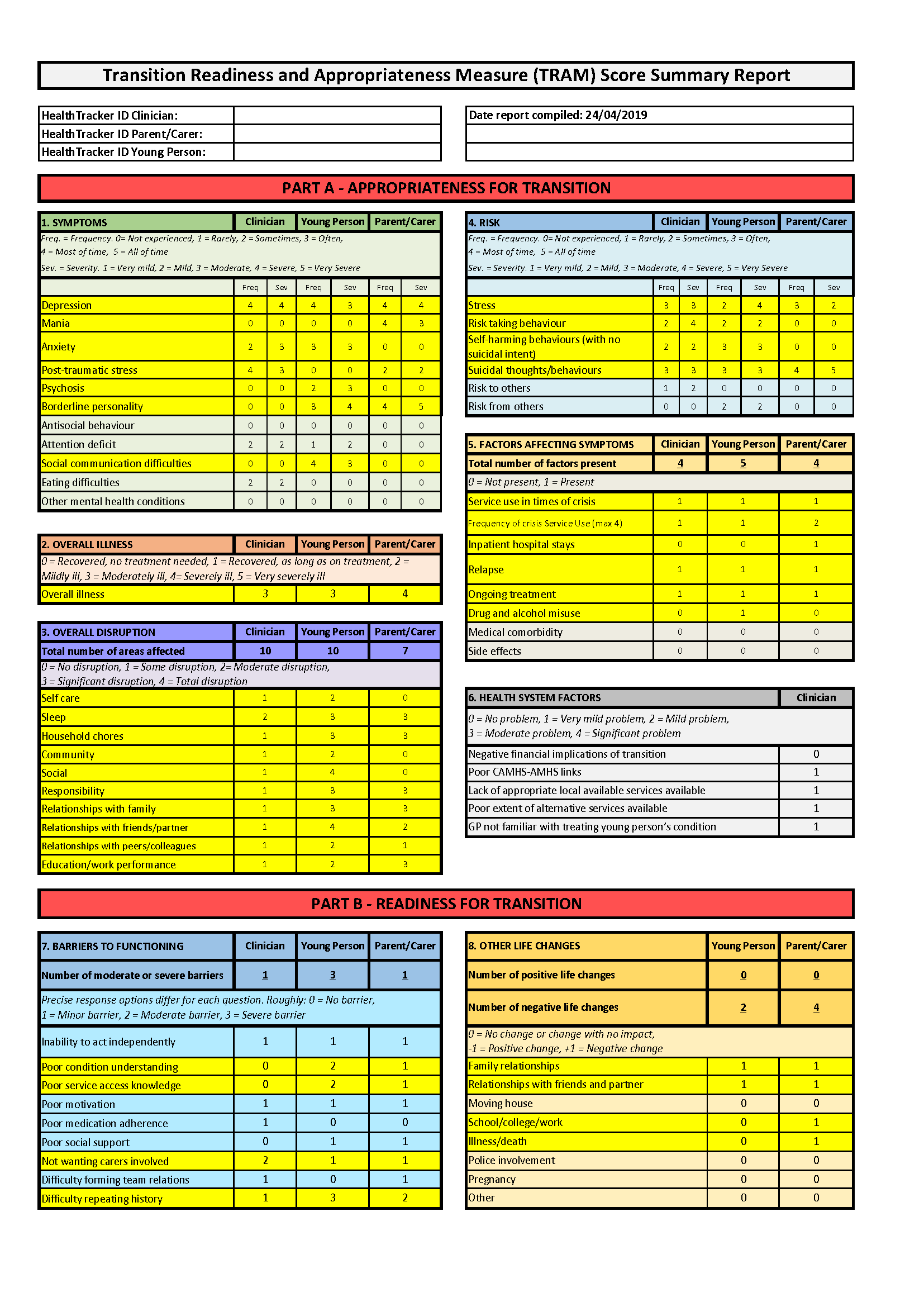 |

## **Figure S3: Email to clinicians that accompanied the TRAM** summary **report**

**Dear Dr Smith,**

We are very grateful for your participation in the MILESTONE study.

Please find attached the **TRAM (Transition Readiness and Appropriateness Measure) score summary report** related to your patient/client [First name, Last name **or** patient’s hospital/clinic ID]. It contains

a) items linked with [his/her] appropriateness for transition to adult mental health services, which are relevant to your transition decision making; and

b) items indicating [his/her] readiness for transition, which may need to be addressed by clinicians on either side of the transition boundary – in order to facilitate a smoother transition.

**Page 1** contains clinician guidance to the score summary report. It explains the data presented in the graphs (PLOTS) and tables on pages 1-2.

**Page 2** presents three plots summarising symptoms, risk factors and overall disruption experienced by the young person as rated by the young person, parent/carer and clinician; and a fourth plot summarising potential barriers to a smooth transition for that YP.

**Page 3** contains all young person, parent/carer and clinician scores from the TRAM in tabular format, with high scores (rated moderate or higher) highlighted in yellow.

In order for you to make the most of the information contained in this report, we recommend the following:

1. Note high scores, or those that stand out, in PLOTS 1-3 as they indicate items that may benefit from consideration when deciding on transition pathways (This information may also be inferred from tables 1, 3 and 4 in Part A).
2. In PLOT 4, note the type and level of potential barriers to successful transition. These are key areas that need to be worked on to ensure successful transition (table 1, Part B).
3. Note the tables that do not have a corresponding graph:
   1. Overall illness (table A2)
   2. Factors that may be affecting symptoms, e.g. drug and alcohol misuse (table A5).
   3. Other life changes (table 2, Part B), which may need consideration when working on potential barriers to a smooth transition.
4. **Note the differences in responses, if any, by the young person, parent/carer and clinician in the graphs and tables.** Differences of opinion are often not spoken out loud and there is potential to address them in a solution-focused way.
5. Use the findings from the TRAM score sheet to focus conversation with your patient/client and their parent/carer on issues surrounding end of care at CAMHS and potential transition to AMHS or other community based service. The TRAM score sheet is confidential so please don’t provide them with copies of it. Instead, use the scores to check understanding of issues and what your patient/client thinks should be done next.
6. Add critical items from the score sheet (items with high score) to a transition plan.
7. Design goals for critical items that are achievable.
8. Include the TRAM summary score sheet in your patient’s/client’s medical notes.
9. If transition to AMHS is decided upon, please include the TRAM summary score report in the referral letter and include our letter to the AMHS clinician (attached to this communication [Appendix 2]). The letter offers the opportunity to discuss the report with one of the MILESTONE team members.

The enclosed “MILESTONE transition leaflet” provides further guidance on factors associated with good quality transition, and contains a frequently asked questions section about TRAM which you may find helpful.

We would like the TRAM score summary report to be as clear to you as possible, so you can make the most of it when making the decision about transition and acting upon it. I am happy to come and explain it further, so please let me know of a date and time most suitable for you. We can also discuss over the phone, if the information in the TRAM summary is unclear or difficult to interpret. Please call me on [tel.no], or email me on [email address] to arrange a suitable time.

I look forward to hearing from you. With best wishes, [MILESTONE research assistant]

## **Figure S4: Transition leaflet that accompanied the TRAM** summary **report**


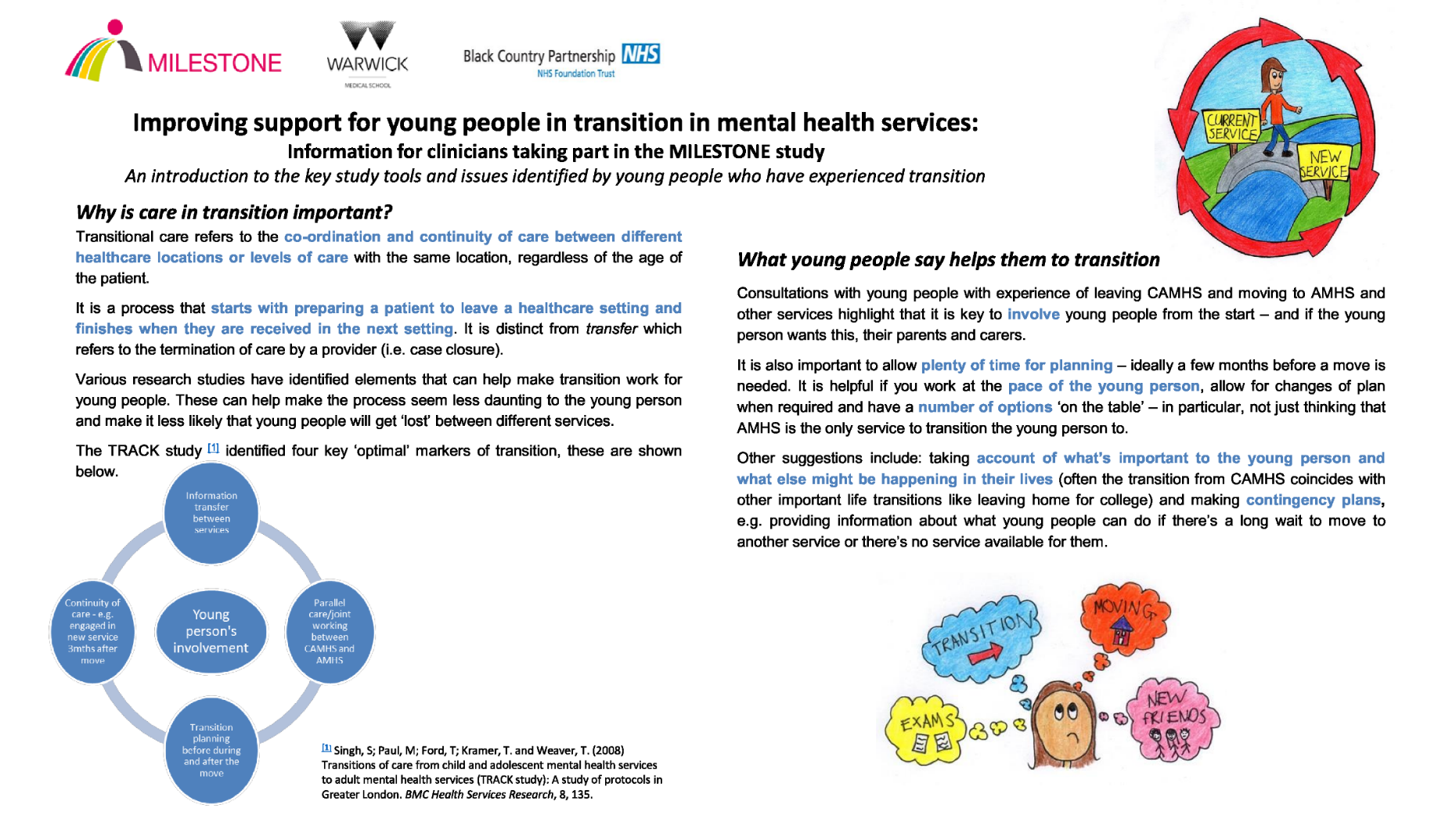


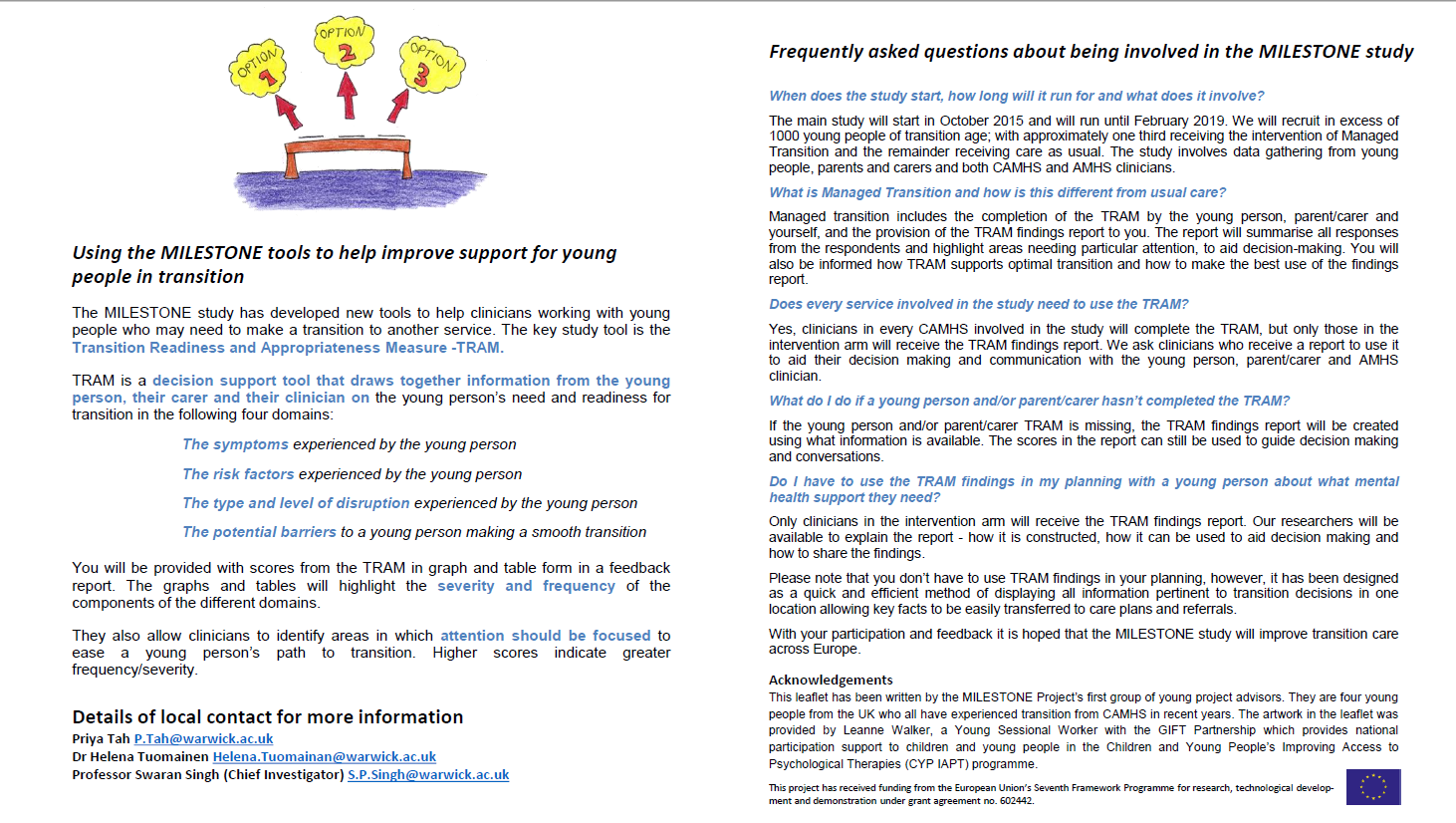


## Figure S5: Recruitment and allocation of clusters


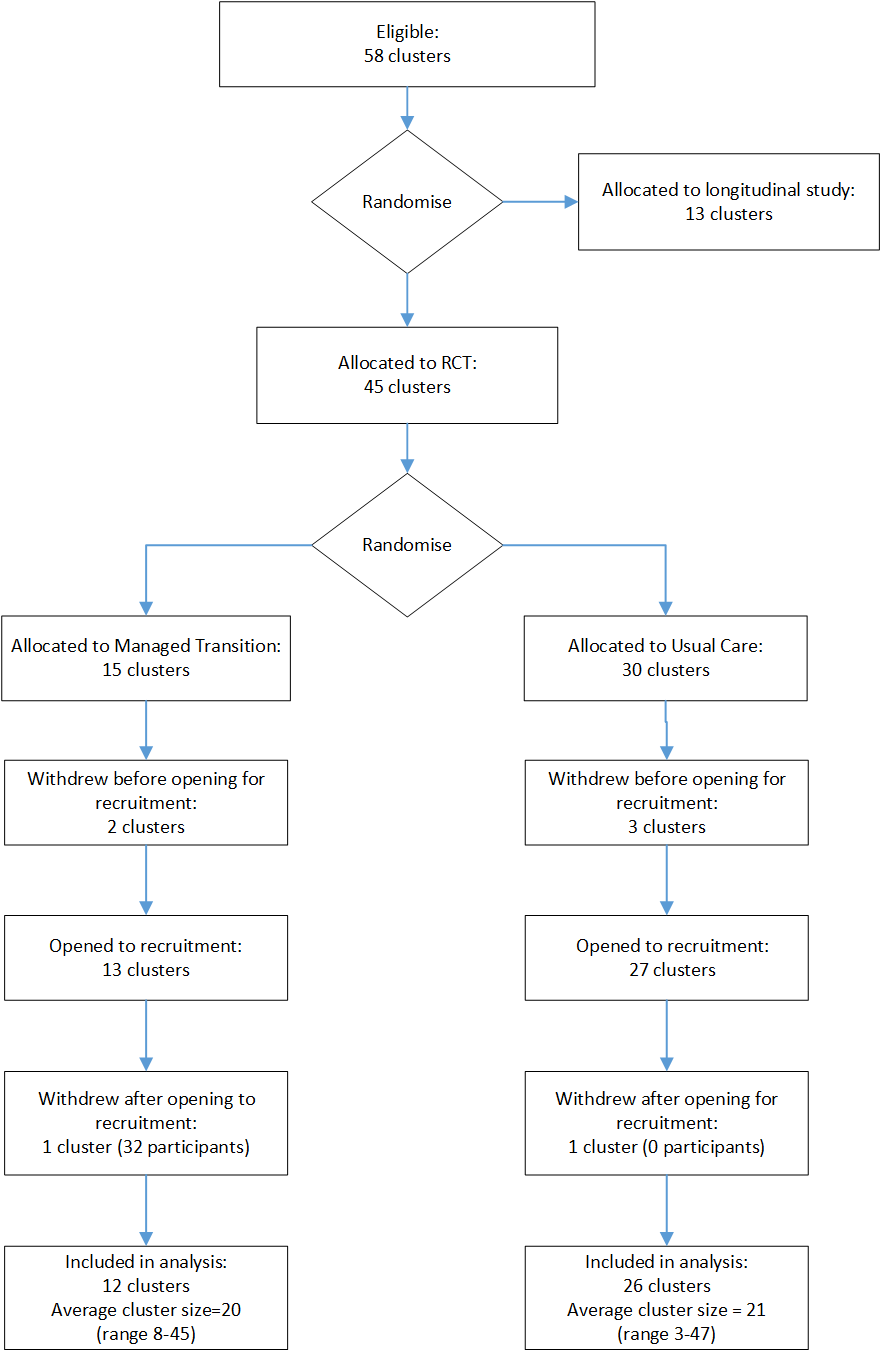


Footnote: For the RCT, 45 clusters were randomised 2:1 between control and intervention. For the combined RCT and longitudinal cohort study (58 clusters), this equates to a randomisation ratio of roughly 3:1 (43 control and 15 intervention). The longitudinal study (not reported here) comprises the 13 clusters directly allocated plus the 30 usual care (control) clusters from the RCT.

## Figure S6: Mean observed HoNOSCA score and standard errors at baseline, 9 months and 15 months

 Footnote: Numbers in the Usual Care group were 529 at baseline, 449 at 9 months and 401 at 15 months and in the Managed Transition group were 229 at baseline, 192 at 9 months and 173 at 15 months.

## Figure S7: Change in observed mean HoNOSCA score since entry to the study


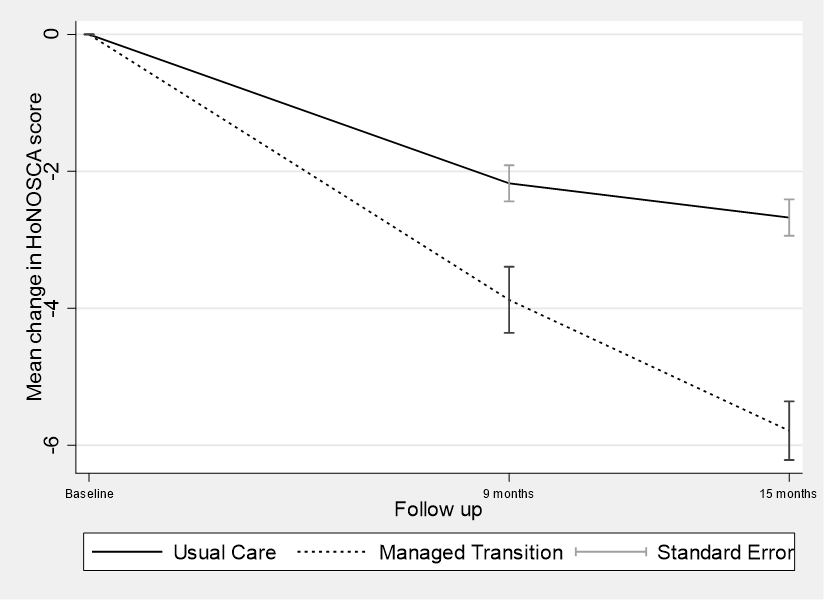


Footnote: Numbers in the Usual Care group were 529 at baseline, 437 at 9 months, and 389 at 15 months and in the Managed Transition group were 229 at baseline, 186 at 9 months, and 168 at 15 months.

## Figure S8: Primary outcome (HoNOSCA score) by pre-planned subgroups

Note: The difference is calculated as Managed Transition minus Usual Care; other diagnoses include substance-related and addictive disorders; schizophrenia spectrum and other psychotic disorders, obsessive-compulsive and related disorders, trauma- and stressor-related disorders, dissociative disorders, somatic symptoms and related disorders, feeding and eating disorders, disorders of adult personality and behaviour, gender dysphoria, other disorders of adult personality and behaviour, relational problems and other circumstances of personal history, unspecified/other mental disorder and multiple primary diagnoses.

## Figure S9: Exploratory analysis of the difference between the trial arms by HoNOSCA domain

Note: The difference is calculated as Managed Transition minus Usual Care.

## Table S1: Baseline characteristics of participants (full version)

|  | **Usual Care**  **(n=552)** | **Managed Transition (n=241)** | **Total (n=793)** |
| --- | --- | --- | --- |
| **Age** (mean, SD) | 17.48 (0.59) | 17.64 (0.51) | 17.53 (0.57) |
| Number (%) age unknown | 5 (0.91%) | 3 (1.25%) | 8 (1.01%) |
| **Gender** (N, %) |  |  |  |
| Female | 340 (61.59%) | 149 (61.83%) | 489 (61.66%) |
| Male | 211 (38.22%) | 90 (37.34%) | 301 (37.69%) |
| Prefer not to say | 1 (0.18%) | 2 (0.83%) | 3 (0.38%) |
| **Ethnicity** (N,%) |  |  |  |
| White | 450 (81.52%) | 210 (87.14%) | 660 (83.23%) |
| Middle Eastern | 1 (0.18%) | 1 (0.41%) | 2 (0.25%) |
| Asian | 5 (0.91%) | 7 (2.90%) | 12 (1.51%) |
| Black/African/Caribbean | 7 (1.27%) | 4 (1.66%) | 11 (1.39%) |
| Central or South American | 6 (1.09%) | 2 (0.83%) | 8 (1.01%) |
| Mixed | 12 (2.17%) | 1 (0.41%) | 13 (1.64%) |
| Unknown/Unspecified | 71 (12.86%) | 16 (6.64%) | 87 (10.97%) |
| **Country** (N, %) |  |  |  |
| Belgium | 64 (11.57%) | 33 (13.69%) | 97 (12.23%) |
| Croatia | 52 (9.42%) | 0 (0.00%)^1^ | 52 (6.56%) |
| France | 66 (11.93%) | 13 (5.39%) | 79 (9.96%) |
| Germany | 64 (11.57%) | 45 (18.67%) | 109 (13.75%) |
| Ireland | 12 (2.17%) | 9 (3.73%) | 21 (2.65%) |
| Italy | 127 (22.97%) | 63 (26.14%) | 190 (23.96%) |
| Netherlands | 75 (13.56%) | 43 (17.84%) | 118 (14.88%) |
| United Kingdom | 92 (16.67%) | 35 (14.52%) | 127 (16.02%) |
| **Primary clinical diagnosis** (N, %) |  |  |  |
| Substance-Related And Addictive Disorders | 3 (0.54%) | 9 (3.73%) | 12 (1.51%) |
| Schizophrenia Spectrum and Other Psychotic Disorders | 26 (4.71%) | 1 (0.41%) | 27 (3.40%) |
| Depressive, Bipolar and Related Disorders | 104 (18.84%) | 60 (24.90%) | 164 (20.68%) |
| Anxiety Disorders | 69 (12.50%) | 25 (10.37%) | 94 (11.85%) |
| Obsessive-Compulsive and Related Disorders | 14 (2.54%) | 4 (1.66%) | 18 (2.27%) |
| Trauma- and Stressor-Related Disorders | 21 (3.80%) | 24 (9.96%) | 45 (5.67%) |
| Dissociative Disorders | 0 (0.00%) | 1 (0.41%) | 1 (0.13%) |
| Somatic Symptoms and Related Disorders | 9 (1.63%) | 2 (0.83%) | 11 (1.39%) |
| Feeding and Eating Disorders | 45 (8.15%) | 9 (3.73%) | 54 (6.81%) |
| Disorders of adult personality and behaviour | 24 (4.35%) | 11 (4.56%) | 35 (4.41%) |
| Gender Dysphoria | 4 (0.72%) | 2 (0.83%) | 6 (0.76%) |
| Other Disorders of adult personality and behaviour | 1 (0.18%) | 0 (0.00%) | 1 (0.13%) |
| Neurodevelopmental Disorders^2^ | 186 (33.70%) | 71 (29.46%) | 257 (32.41%) |
| Other^3^ | 3 (0.54%) | 1 (0.41%) | 4 (0.50%) |
| Unspecified/Other Mental Disorder | 2 (0.36%) | 2 (0.83%) | 4 (0.50%) |
| Multiple primary diagnoses | 21 (3.80%) | 9 (3.73%) | 30 (3.78%) |
| None | 20 (3.62%) | 10 (4.15%) | 30 (3.78%) |
| **Socioeconomic status** (N, %) |  |  |  |
| **Highest completed education level** |  |  |  |
| Primary | 218 (39.49%) | 82 (34.02%) | 300 (37.83%) |
| Secondary | 277 (50.18%) | 123 (51.04%) | 400 (50.44%) |
| Further/higher education | 8 (1.45%) | 6 (2.49%) | 14 (1.77%) |
| Other/Vocational training | 4 (0.72%) | 2 (0.83%) | 6 (0.76%) |
| Unknown | 45 (8.15%) | 28 (11.62%) | 73 (9.21%) |
| **Employment status** |  |  |  |
| Paid or self-employment | 66 (11.96%) | 40 (16.60%) | 106 (13.37%) |
| Voluntary employment | 8 (1.45%) | 6 (2.49%) | 14 (1.77%) |
| Unemployed | 68 (12.32%) | 33 (13.69%) | 101 (12.74%) |
| Student | 318 (57.61%) | 124 (51.45%) | 442 (55.74%) |
| Other | 12 (2.17%) | 9 (3.73%) | 21 (2.65%) |
| Unknown | 80 (14.49%) | 29 (12.03%) | 109 (13.75%) |
| **Usual living situation** (N, %) |  |  |  |
| Both biological parents | 298 (53.99%) | 113 (46.89%) | 411 (51.83%) |
| One biological parent only | 165 (29.89%) | 67 (27.80%) | 232 (29.26%) |
| Adoptive or foster parents | 1 (1.45%) | 9 (3.73%) | 17 (2.14%) |
| Living independently | 5 (0.91%) | 6 (2.49%) | 11 (1.39%) |
| Living in residential care | 18 (3.26%) | 24 (9.96%) | 42 (5.30%) |
| Other | 20 (3.62%) | 11 (4.56%) | 31 (3.91%) |
| Unknown | 38 (6.88%) | 11 (1.56%) | 49 (6.18%) |
| **Time spent under CAMHS care** (N, %) |  |  |  |
| Less than 6 months | 52 (9.42%) | 43 (17.84%) | 95 (11.98%) |
| 6 months to 1 year | 90 (16.30%) | 42 (17.43%) | 132 (16.65%) |
| 1 year to 2 years | 91 (16.49%) | 30 (12.45%) | 121 (15.26%) |
| 2 years to 5 years | 145 (26.27%) | 57 (23.65%) | 202 (25.47%) |
| 5 to 10 years | 76 (13.77%) | 37 (15.35%) | 113 (14.25%) |
| More than 10 years | 49 (8.88%) | 17 (7.05%) | 66 (8.32%) |
| Unknown | 49 (8.88%) | 15 (6.22%) | 64 (8.07%) |
| **Parent/Carer (P/C) involved in MILESTONE** (N, %) |  |  |  |
| Yes | 474 (85.87%) | 194 (80.50%) | 668 (84.24%) |
| No | 78 (14.13%) | 47 (19.50%) | 125 (15.76%) |
| **HoNOSCA** (mean, SD) | 11.60 (6.86) | 13.78 (7.11) | 12.14 (6.98) |
| Number missing (%) | 23 (4.17%) | 12 (4.98%) | 35 (4.41%) |
| **HoNOSCA Self Report (SR)** (mean, SD) | 12.26 (9.23) | 12.72 (8.33) | 12.40 (8.96) |
| Number missing (%) | 39 (7.07%) | 15 (6.22%) | 54 (6.81%) |
| **TRAM (Clinician report)** |  |  |  |
| Subscales (mean, SD): |  |  |  |
| Symptoms | 17.74 (9.97) | 21.23 (9.95) | 18.85 (10.09) |
| Risk | 14.70 (12.50) | 20.11 (13.06) | 16.42 (12.92) |
| Overall Disruption | 24.89 (18.40) | 26.28 (15.97) | 25.33 (17.67) |
| Factors Affecting Symptoms | 23.86 (18.59) | 27.40 (20.26) | 24.99 (19.20) |
| Barriers to Functioning | 26.12 (14.20) | 27.77 (13.58) | 26.64 (14.02) |
| Overall Illness (takes into account all symptoms across all existing conditions) (N, %): |  |  |  |
| Recovered – ongoing treatment not required | 59 (10.69%) | 24 (9.96%) | 83 (10.47%) |
| Recovered – symptoms absent as long as on treatment | 80 (14.49%) | 22 (9.13%) | 102 (12.86%) |
| Mildly ill | 102 (18.48%) | 45 (18.67%) | 147 (18.54%) |
| Moderately ill | 142 (25.72%) | 84 (34.85%) | 226 (28.50%) |
| Severely ill | 77 (13.95%) | 41 (17.01%) | 118 (14.88%) |
| Very severely ill | 7 (1.27%) | 3 (1.24%) | 10 (1.26%) |
| Unknown | 85 (15.40%) | 22 (9.13%) | 107 (13.49%) |
| Number (%) YP with no TRAM CR at T1 | 85 (15.40%) | 22 (9.13%) | 107 (13.49%) |
| **WHO Quality of Life Brief Inventory (WHOQOL-BREF)** (mean, SD) |  |  |  |
| Physical health | 14.84 (2.66) | 14.41 (2.93) | 14.71 (2.75) |
| Psychological | 12.20 (3.56) | 11.83 (3.57) | 12.09 (3.57) |
| Social relationships | 13.72 (3.31) | 13.99 (3.36) | 13.80 (3.33) |
| Environment | 15.15 (2.64) | 14.86 (2.52) | 15.06 (2.60) |
| Number (%) missing | 48 (8.70%) | 26 (10.79%) | 74 (9.33%) |
| **EQ-5D-5L (health status)** (mean, SD) | 0.78 (0.20) | 0.78 (0.20) | 0.78 (0.20) |
| Number (%) missing | 47 (8.51) | 25 (10.37) | 72 (9.08) |
| **Specific Levels of Functioning Scale (SLOF)** (median, IQR) |  |  |  |
| Physical Functioning | 25 (24-25) | 25 (24-25) | 25 (24-25) |
| Personal Care Skills | 35 (33-35) | 34 (33-35) | 35 (33-35) |
| Interpersonal Relationships | 27 (21-32) | 27 (22-32) | 27 (21-32) |
| Social Acceptability | 33 (30-35) | 31 (29-34) | 32 (30-34) |
| Activities | 52 (48-54) | 52 (49-54) | 52 (48-54) |
| Work Skills | 25 (20-29) | 25 (20-28) | 25 (20-29) |
| Number (%) missing | 132 (23.91%) | 68 (28.21%) | 200 (25.22%) |
| **Youth Self Report (YSR), Adult Self Report (ASR)** (t-scores) (mean, SD) |  |  |  |
| Internalising problems | 60.62 (12.56) | 63.42 (12.73) | 61.47 (12.67) |
| Externalising problems | 51.54 (10.61) | 53.64 (9.67) | 52.18 (10.37) |
| Number (%) missing | 55 (9.96) | 24 (9.96) | 79 (9.96) |
| **Child Behaviour Checklist (CBCL), Adult Behaviour Checklist (ABCL)** (t-scores)(mean, SD) |  |  |  |
| Internalising problems | 63.62 (10.90) | 65.81 (10.42) | 64.24 (10.80) |
| Externalising problems | 53.75 (10.23) | 57.15 (10.67) | 54.72 (10.47) |
| Number (%) missing | 137 (24.82) | 75 (31.12 | 212 (26.73) |
| **Independent Behaviour During Consultation Scale** (mean, SD) | 13.09 (6.43) | 15.11 (6.14) | 13.70 (6.41) |
| Number (%) missing | 56 (10.14%) | 27 (11.20%) | 83 (10.47%) |
| **Severity of illness (CGI)** |  |  |  |
| Not assessed (0) | 13 (2.36%) | 5 (2.07%) | 18 (2.27%) |
| Normal, not at all ill (1) | 33 (5.98%) | 12 (4.98%) | 45 (5.67%) |
| Borderline mentally ill (2) | 75 (13.59%) | 30 (12.45%) | 105 (13.24%) |
| Mildly ill (3) | 114 (20.65%) | 44 (18.26%) | 158 (19.92%) |
| Moderately ill (4) | 128 (23.19%) | 70 (29.05%) | 198 (24.97%) |
| Markedly ill (5) | 73 (13.22%) | 45 (18.67%) | 118 (14.88%) |
| Severely ill (6) | 34 (6.16%) | 11 (4.56%) | 45 (5.67%) |
| Among the most extremely ill patients (7) | 6 (1.09%) | 3 (1.24%) | 9 (1.13%) |
| Unknown | 76 (13.77%) | 21 (8.71%) | 97 (12.23%) |

^1^ We randomised three clusters from Croatia (2 to the UC arm and one to MT) but had to withdraw one of the clusters (MT arm) from the study due to uncertainty regarding the validity of participant consent. The data collected from this site is therefore excluded from the analysis; ^2^ Includes 42 with specific learning disorders (36 UC, 6 MT), 74 with autism (56 UC, 18 MT) and 97 with ADHD (69 UC, 28 MT).^3^ Includes relational problems and other circumstances of personal history.

## Table S2: Characteristics of participating CAMHS (clusters)

| **Country** | **Site** | **Service boundary**  **(age in years)** | **Written transition policy in place? (Y/N)** | **Type of service** |
| --- | --- | --- | --- | --- |
| Belgium | Antwerp  Brugge  Brussel  Genk  Leuven  Kortenberg | 18  18  18  18  18  18 | Unknown | Hospital based  Hospital based  Community based  Community and Hospital based  Hospital based  Community and Hospital based |
| Croatia | Osijek  Split  Zagreb | 18  18  N/A | N/A | Other  Other  Withdrew |
| France | Marseille  Montpellier  Tours | 18  18  17 | Y | Community and Hospital based  Community and Hospital based, Other  Hospital based |
| Germany | Augsburg  Ravensburg  Ulm | 18  18  18 |  | Hospital based  Hospital based  Hospital based |
| Italy | Bari  Brescia  Lecco  Milano SP  Modena  Monza  Padova  Parma  Roma^2^ | 18  18  18  18  18  18  18  18  18 | Y  Y  Y  Y  Y | Hospital based  Community and Hospital based  Community and Hospital based  Community and Hospital based  Community and Hospital based, Other  Community and Hospital based  Community and Hospital based  Community based, Other  Hospital based |
| Netherlands | Erasmus MC  GGZ WNB^1^  Lucertis  Mondriaan  Virenze  Yulius B | 18  18  18  18  18  18 | Y | Hospital based  Community based  Community and Hospital based  Community based, Other  Community based  Community based |
| Republic of Ireland | North Cork  Roscommon/East Galway  South Lee/ West Cork | 18  N/A  18 |  | Community based  Community based (Withdrew)  Community based |
| United Kingdom | Dudley  Lambeth  Lewisham  North Worcestershire  South Staffordshire and Shropshire  Southwark  Wolverhampton | 16  18  18  18  18  18  18 | Y  Y  Y  Y | Community based  Community and Hospital based  Community and Hospital based  Community based  Community based  Community and Hospital based  Community based |

^1^ Geestelijke Gezondheidszorg Westelijk Noord-Brabant

^2^ Privately funded, all other government and/or health insurance funded

## Table S3: Schedule of data collection

| **Follow Up** | **Baseline** | **9 months** | **15 months** |
| --- | --- | --- | --- |
| **Contact Window** | **Within 6m before Service Boundary** | **9m (± 1m) after Baseline** | **15m (± 1m) after Baseline** |
| Sociodemographic & personal information | **YP**  **P/C**  **C** | **YP**  **(C)** | **YP**  **P/C**  **(C)** |
| Need for Care  (HoNOSCA – Self Report)  (HoNOSCA – Clinician Rated) | **YP**  **RA** | **YP**  **RA** | **YP**  **RA** |
| Transition readiness / Transition outcome (TRAM/TROM) | **YP**  **P/C**  **C** | **YP**  **P/C**  **C** | **YP**  **P/C**  **C** |
| Referral and Transition Status (CAMHS clinician only) |  | **C** | **(C)** |
| Quality of Life (WHOQOL-BREF) | **YP** |  | **YP** |
| Cost-effectiveness (EQ-5D-5L) | **YP** | **YP** | **YP** |
| Service use (CSRI) | **YP** | **YP** | **YP** |
| Emotional/behavioural problems  YSR/ASR  CBCL/ABCL | **YP**  **P/C** | **YP**  **P/C** | **YP**  **P/C** |
| Independent behaviour*  (IBDCS/BtC) | **YP** | **YP** | **YP** |
| Transition experience & readiness (OYOF)* |  | **YP**  **P/C** | **(YP)**  **(P/C)** |
| Functioning & Impairment  (SLOF) | **P/C** |  | **P/C** |
| Illness severity (CGIS) | **C** | **(C)** | **(C)** |
| Psychopathology  C: Clinical diagnosis | **C** | **(C)** | **(C)** |

YP = young person; P/C = parent/carer; C = clinician; (C) = for YP who are mental health service users only; RA = Research Assistant; * IBDCS if YP is a mental health service user, BtC is completed;**completed only once at the first follow up post- transition

## Table S4: List of scales used and their purpose

| **Variable** | **Description** |
| --- | --- |
| HONOSCA | The Health of the Nation Outcome Scale for Children and Adolescents (HoNOSCA) measures the range of physical, personal and social problems associated with mental illness, providing an indication of need for care. The 15 item questionnaire indicates the severity of each problem on a scale of 0-4. The 13 main HoNOSCA items (generally summed to give a total score) cover: Behaviour, Impairment, Symptoms and Social Functioning. Two further items cover lack of knowledge about the nature and management of child’s difficulties. |
| TRAM | The Transition Readiness and Appropriateness Measure (TRAM) identifies the needs, preferences and readiness of young people for ongoing adult care, including those who can be appropriately discharged or transitioned to other service. TRAM has three versions (adolescent, parent/carer, and clinician) and includes 74 items on Functioning related to the transition process, Satisfaction with service, Recent life changes, Symptoms frequency and severity, Overall illness, Overall disruption, and Risk factors. |
| TROM | The Transition Related Outcome Measure (TROM) assesses the transition process, experience and outcomes, with versions for young person, parent/carer and clinician. The TROM mirrors the TRAM and contains the following subscales Symptoms Frequency, Symptoms Change Severity, Risk Factors Frequency, Risk Factors Change Severity, Overall disruption, Activities done that facilitated transition. |
| WHOQOL-BREF | World Health Organization Quality of Life Brief Inventory (WHOQOL-BREF). Quality of life will be assessed using the self-reported WHOQOL-BREF. It provides a short form (26 items) quality of life assessment with a cross-cultural perspective that covers self-reported quality of life on physical and psychological health, social relationships and current environment. |
| SLOF | Specific Levels of Functioning (SLOF) Scale will assess the broader domain of adult functioning, the SLOF will be used to assess adult functioning from the perspective of the parent/carer. The SLOF was developed to assess an individual’s social functioning and everyday living skills and consists of 43 behavioural items. |
| ASEBA YP  (YSR, ASR) | The Achenbach System of Empirically Based Assessment (ASEBA) assesses the dimensions of emotional and behavioural problems. Young people (YP) will complete the Youth Self Report (YSR) or the Adult Self Report (ASR) for those aged 18+. |
| ASEBA PC  (CBCL, ABCL) | The Achenbach System of Empirically Based Assessment (ASEBA) assesses the dimensions of emotional and behavioural problems. Parents/carers (P/C) provide information on their children’s emotional and behavioural problem using the Child Behaviour Checklist or Adult Behaviour Checklist for those aged 18+. |
| IBDCS | Independent Behaviour During Consultation Scale (IBDCS). The IBDCS is a 7-item scale obtained from an instrument measuring adolescents’ self-efficacy (on a 5-point Likert scale) which also included a scale for measuring self-efficacy in skills for independent hospital visits. |
| CGIS | Clinical Global Impression – Severity (CGIS) is an observer-rated one item scale that measures illness severity on a 7-point scale. It requires the clinician to rate the severity of the patient's illness at the time of assessment, relative to the clinician's past experience with patients who have the same diagnosis. |
| OYOF-TES | On Your Own Feet The Transition Experience Scale (OYOF-TES) consists of 18 items rated on ﬁve-point Likert scales. The items focus on specific experiences with the transfer process. There are two versions of the scale: one for young people who are referred to AMHS, and one for young people discharged from CAMHS (i.e. not making transition to AMHS). |
| EQ-5D-5L | EQ-5D-5L is a standardised instrument for use as a measure of health outcome. It provides a simple descriptive profile and a single index value for health status. The EQ-5D will be scored using the devised algorithms and summarised according to the User Guide. |
| CSRI | The Client Service Receipt Inventory (CSRI) focuses on health and social service use. It has been adapted from a scale used in a European research project on schizophrenia, which was effective in tracing patterns of service use in an international population and made comparison among different countries possible. |

## Table S5: Baseline characteristics of parent/carers

|  | **Usual Care (N=472)** | **Managed Transition**  **(N=194)** | **Total**  **(N=666)** |
| --- | --- | --- | --- |
| Age (mean, SD) | 47.87 (6.09)  (N=130) | 48.54 (8.02)  (N=40) | 48.02 (6.57)  (N=171) |
| Gender  Female (N, %)  Male (N,%)  Prefer not to say (N,%)  Missing (N,%) | 366 (77.54%)  73 (15.47%)  31 (6.57%)  2 (0.42%) | 159 (81.96%)  25 (12.89%)  3 (1.55%)  7 (3.61%) | 525 (78.83%)  98 (14.71%)  34 (5.11%)  9 (1.35%) |
| Ethnicity (N, %)  White  Middle Eastern  Asian  Black  Central or South American  Mixed  Other  Not known | 361 (76.48%)  1 (0.21%)  4 (0.85%)  0 (0.00%)  3 (0.64%)  4 (0.85%)  0 (0.00%)  99 (20.97%) | 147 (75.77%)  0 (0.00%)  3 (1.55%)  0 (0.00%)  1 (0.52%)  0 (0.00%)  0 (0.00%)  43 (22.16%) | 508 (76.28%)  1 (0.15%)  7 (1.05%)  0 (0.00%)  4 (0.60%)  4 (0.60%)  0 (0.00%)  142 (21.32%) |
| Relationship to participant (N, %)  Parent  Grandparent  Sibling  Other family member  Carer  Unknown | 457 (96.82%)  2 (0.42%)  1 (0.21%)  2 (0.42%)  8 (1.69%)  2 (0.42%) | 173 (89.18%)  2 (1.03%)  1 (0.52%)  2 (1.03%)  9 (4.64%)  7 (3.61%) | 630 (94.59%)  4 (0.60%)  2 (0.30%)  4 (0.60%)  17 (2.55%)  9 (1.35%) |
| Country (N, %)  Belgium  Croatia  France  Germany  Italy  Netherlands  Republic of Ireland  United Kingdom | 48 (10.17%)  52 (11.02%)  62 (13.14%)  61 (12.92%)  120 (25.42%)  64 (13.56%)  12 (2.54%)  53 (11.23%) | 25 (12.89%)  0 (0.00%)  13 (6.70%)  40 (20.62%)  58 (29.90%)  33 (15.46%)  9 (4.64%)  29 (9.79%) | 73 (10.96%)  52 (7.81%)  75 (11.26%)  101 (15.17%)  178 (26.73%)  97 (14.11%)  21 (3.15%)  72 (10.81%) |
| Socioeconomic status (education level) (N, %)  Primary  Secondary  Tertiary  University  Other  Missing | 43 (9.11%)  149 (31.57%)  97 (20.55%)  114 (24.15%)  17 (3.60%)  52 (11.02%) | 34 (17.53%)  60 (30.93%)  46 (23.16%)  44 (22.68%)  3 (1.55%)  10 (5.16%) | 77 (11.56%)  209 (31.38%)  140 (21.02%)  158 (23.72%)  20 (3.00%)  62 (9.31%) |

## Table S6: Characteristics of CAMHS clinicians

|  | **Usual Care**  **(N=198)** | **Managed Transition**  **(N=95)** | **Total**  **(N=293)** |
| --- | --- | --- | --- |
| Country (N, %)  Belgium  Croatia  France  Germany  Italy  Netherlands  Republic of Ireland  United Kingdom | 30 (15.15%)  6 (3.03%)  12 (6.06%)  34 (17.17%)  30 (20.95%)  48 (24.24%)  2 (1.01%)  36 (18.18%) | 14 (14.74%)  0 (0.00%)  3 (3.16%)  13 (13.68%)  14 (14.74%)  31 (32.63%)  2 (2.11%)  18 (18.95%) | 44 (15.02%)  6 (2.05%)  15 (5.12%)  47 (16.04%)  44 (15.02%)  79 (26.96%)  4 (1.37%)  54 (18.43%) |
| Age group (years)  Below 25  25-35  35-45  45-55  55-65  65+  Prefer not to say | 3 (1.52%)  53 (26.77%)  70 (35.35%)  54 (27.27%)  17 (8.59%)  0 (0.00%)  1 (0.51%) | 4 (4.21%)  22 (23.16%)  30 (31.58%)  17 (17.89%)  21 (22.11%)  1 (1.05%)  0 (0.00%) | 7 (2.39%)  75 (25.60%)  100 (34.13%)  71 (24.23%)  38 (12.97%)  1 (0.34%)  1 (0.34%) |
| Gender (N, %)  Female  Male  Prefer not to say | 281 (70.07%)  118 (29.43%)  2 (0.50%) | 136 (71.96%)  53 (28.04%)  0 (0.00%) | 417 (70.68%)  171 (28.98%)  2 (0.34%) |
| Experience of working in mental health (years) (N,%)  0-3  3-5  5-10  10-15  15-20  20-25  25+ | 20 (10.10%)  22 (11.11%)  37 (18.69%)  49 (24.75%)  29 (14.65%)  29 (14.65%)  12 (6.06%) | 5 (5.26%)  7 (7.37%)  20 (21.05%)  19 (20.00%)  10 (10.53%)  12 (12.63%)  22 (23.16%) | 25 (8.53%)  29 (9.90%)  57 (19.45%)  68 (23.21%)  39 (13.31%)  41 (13.99%)  34 (11.60%) |
| Professional group (N, %)  Psychologist  Psychiatrist  Nurse  Psychotherapist  Family therapist  Support worker  Other | 51 (25.76%)  88 (44.44%)  16 (8.08%)  28 (14.14%)  5 (2.53%)  1 (0.51%)  9 (4.55%) | 38 (40.00%)  22 (23.16%)  3 (3.16%)  21 (22.11%)  5 (5.26%)  1 (1.05%)  5 (5.26%) | 89 (30.38%)  110 (37.54%)  19 (6.48%)  49 (16.72%)  10 (3.41%)  2 (0.68%)  14 (4.78%) |

Note: Numbers relate to 590 participants (401 in the UC arm and 189 in the MT arm). We were unable to obtain information regarding the CAMHS clinician for 203 (25.60%) young people at baseline (52/241 (21.57%) in the managed transition arm and 151/552 (27.36%) in the Usual Care arm). It is likely that a large proportion of these are new cases where a clinician is yet to be assigned.

## Table S7: Characteristics of AMHS clinicians

|  | **Usual Care**  **(N=55)** | **Managed Transition**  **(N=58)** | **Total**  **(N=113)** |
| --- | --- | --- | --- |
| Country (N, %)  Belgium  Croatia  France  Germany  Italy  Netherlands  Republic of Ireland  United Kingdom | 5 (9.09%)  1 (1.82%)  11 (20.00%)  7 (12.73%)  20 (36.36%)  7 (12.73%)  0 (0.00%)  4 (7.27%) | 6 (10.34%)  0 (0.00%)  2 (3.45%)  1 (1.72%)  48 (82.76%)  0 (0.00%)  0 (0.00%)  1 (1.72%) | 11 (9.73%)  1 (0.88%)  13 (11.50%)  8 (7.08%)  68 (60.18%)  7 (6.19%)  0 (0.00%)  5 (3.54%) |
| Age group (years)  Below 25  25-35  35-45  45-55  55-65  65+  Prefer not to say | 1 (1.82%)  12 (21.82%)  10 (18.18%)  22 (40.00%)  7 (12.73%)  1 (1.82%)  2 (3.64%) | 0 (0.00%)  5 (8.62%)  25 (43.10%)  21 (36.21%)  6 (10.34%)  0 (0.00%)  1 (1.72%) | 1 (0.88%)  17 (15.04%)  35 (30.97%)  43 (38.05%)  13 (11.50%)  1 (0.88%)  3 (2.65%) |
| Gender (N, %)  Female  Male | 34 (61.82%)  21 (38.18%) | 45 (77.59%)  13 (22.41%) | 79 (69.91%)  34 (30.09%) |
| Experience of working in mental health (years) (N, %)  0-3  3-5  5-10  10-15  15-20  20-25  25+  Prefer not to say | 3 (5.45%)  8 (14.55%)  12 (21.82%)  5 (9.09%)  10 (18.18%)  9 (16.36%)  7 (12.73%)  1 (1.82%) | 3 (5.17%)  0 (0.00%)  1 (1.72%)  29 (50.00%)  10 (17.24%)  13 (22.41%)  1 (1.72%)  1 (1.72%) | 6 (5.31%)  8 (7.08%)  13 (11.50%)  34 (30.09%)  20 (17.70%)  22 (19.47%)  8 (7.08%)  2 (1.77%) |
| Professional group (N, %)  Psychologist  Psychiatrist  Nurse  Psychotherapist  Support worker | 10 (18.18%)  37 (67.27%)  2 (3.64%)  5 (9.09%)  1 (1.82%) | 15 (25.86%)  41 (70.69%)  1 (1.72%)  1 (1.2%)  0 (0.00%) | 25 (22.12%)  78 (67.03%)  3 (2.65%)  6 (5.31%)  1 (0.88%) |

Note: Numbers may include some non-AMHS mental health practitioners and relate to 76 young people (43 in the usual care arm and 33 in the managed transition arm). It was difficult to for the trial team to engage with clinicians in AMHS and other adult mental health services. Information on AMHS clinicians may therefore be missing either because the young person did not transition to AMHS (they were discharged to a GP, for example) or because we were unable to contact them.

## Table S8: Transition decisions and referrals by CAMHS clinicians

|  | Usual Care  (N=552) | Managed Transition  (N=241) | Total  (N=793) |
| --- | --- | --- | --- |
| Has the young person been referred to another service? (N, %)  Not yet referred/still under original CAMHS care  Referred to another CAMHS  Referred to AMHS  Referred to other service provider  Discharged to GP  Discharged from care with no referral  Unknown | 151 (27.36%)  20 (3.62%)  83 (15.04%)  51 (9.24%)  45 (8.15%)  86 (15.58%)  116 (21.01%) | 59 (24.48%)  17 (7.05%)  48 (19.92%)  14 (5.81%)  27 (11.20%)  34 (14.11%)  42 (17.43%) | 210 (26.48%)  37 (4.67%)  131 (16.52%)  65 (8.20%)  72 (9.08%)  120 (15.13%)  158 (19.92%) |
| Have the TRAM findings been shared with the young person?^1^ (N, %)  Yes  No  Not yet, but has plans to do so  Unknown  Not applicable | 15 (2.72%)  9 (1.63%)  4 (0.72%)  94 (17.03%)  430 (77.90%) | 69 (28.63%)  97 (40.25%)  16 (6.64%)  55 (22.82%)  4 (1.66%) | 84 (10.59%)  106 (13.37%)  20 (2.52%)  149 (18.79%)  434 (54.73%) |
| What advice did you give the young person regarding transition? (N, %)  Continue in CAMHS  Transition to AMHS  Continue in other service  Discharge to GP  End of care  Not yet decided  Unknown | 138 (25.00%)  116 (21.01%)  59 (10.69%)  48 (8.70%)  65 (11.78%)  2 (0.36%)  124 (22.64%) | 69 (28.63%)  65 (26.97%)  17 (7.05%)  21 (8.71%)  28 (11.62%)  0 (0.00%)  41 (17.01%) | 207 (26.10%)  181 (22.82%)  76 (9.58%)  69 (8.70%)  93 (11.73%)  2 (0.25%)  165 (20.81%) |
| Was the young person accepted by the new service? (N, %)  No referral, still in same CAMHS  Accepted and under care of new service  Accepted but care has not started yet  Rejected by service referred to  Discharged with no referral  Unknown | 150 (27.17%)  122 (22.10%)  15 (2.72%)  3 (0.54%)  86 (15.58%)  176 (31.88%) | 59 (24.48%)  68 (28.22%)  3 (1.24%)  3 (1.24%)  34 (14.11%)  74 (30.71%) | 209 (26.36%)  190 (23.96%)  18 (2.27%)  6 (0.76%)  120 (15.13%)  250 (31.53%) |
| Timing of transition decision (N, %)  0-9 months after entry to trial  9-15 months after entry to trial  No decision made (still in CAMHS at 15 months)  Unable to obtain information | 275 (49.82%)  37 (7.07%)  116 (20.65%)  124 (22.46%) | 126 (52.28%)  21 (8.71%)  53 (21.99%)  41 (17.01%) | 401 (50.57%)  58 (7.31%)  169 (21.31%)  165 (20.81%) |

^1^ Clinicians in the MT group were advised to use the TRAM findings to focus the transition discussion but there was no specific requirement to share the TRAM findings the young person.

## Table S9: Number, nature, severity and **relatedness** of severe adverse events (SAE) in each treatment group

|  | Usual Care | Managed Transition | Total |
| --- | --- | --- | --- |
| No of SAEs reported^1,2^ | 25 | 5 | 30 |
| **No of participants experiencing an SAE** | **18** | **4** | **22** |
|  |  |  |  |
| **Fatal/Life-threatening** | **13** | **2** | **15** |
| Death^2^ | *0* | *1* | *1* |
| *Suicide attempt* | *11* | *1* | *12* |
| *Hospital admission due to alcohol intoxication* | *2* | *0* | *2* |
|  |  |  |  |
| **Severe** | **9** | **2** | **11** |
| *Suicide attempt* | *7* | *2* | *9* |
| *Suicidal thoughts* | *0* | *0* | *0* |
| *Hospital admission due to alcohol intoxication* | *1* | *0* | *1* |
| *Hospital admission (not mental health related)* | *1* | *0* | *1* |
|  |  |  |  |
| **Moderate** | **3** | **1** | **4** |
| *Suicide attempt* | *0* | *1* | *1* |
| *Suicidal thoughts^3^* | *1* | *0* | *1* |
| *Risk of self-harm^3^* | *1* | *0* | *1* |
| *Hospital admission (not mental health related)* | *1* | *0* | *1* |

^1^ Participants may experience more than one SAE. ^2^ None of the SAEs are believed to be related to the intervention or participation in the trial. ^3^ Includes those reported as an SAE only.

## Table S10: Intervention costing example – UK

| **UK TRAM Intervention Delivery Costs** | | | | | | | **UK Training costs** | | |
| --- | --- | --- | --- | --- | --- | --- | --- | --- | --- |
| TRAM completion time | | TRAM report creation | | TRAM feedback | | **Total Delivery costs per UK intervention child (Euros)** | Clinician training costs | | |
| Mean TRAM completion time UK (hours) | Cost per UK intervention child (Euros) | TRAM report generation admin (hrs) | TRAM report admin cost per UK intervention child (Euros) | Tram Feedback UK per child (hrs) | Tram feedback cost per intervention child |  | Training hours - UK | Total Training costs - UK | **Clinician training cost per UK intervention Child (Euros)** |
| 0.22 | 27.02 | 0.28 | 3.12 | 0.2 | 24.94 | **55.08** | 45.9 | 5724.65 | **163.56** |
